# Different evolutionary patterns of *TIR1/AFBs* and *AUX/IAAs* and their implications for the morphogenesis of land plants

**DOI:** 10.1186/s12870-023-04253-4

**Published:** 2023-05-19

**Authors:** Liyao Su, Tian Zhang, Bin Yang, Tianyu Dong, Xiaoyu Liu, Yibo Bai, Hui Liu, Jingsong Xiong, Yan Zhong, Zong-Ming (Max) Cheng

**Affiliations:** State Key Laboratory of Crop Genetics and Germplasm Enhancement, College of Horticulture, Nanjing Agricultural University, Nanjing, 210095 China

**Keywords:** Auxin, TIR1/AFB, AUX/IAA, Interactions pattern, Gene retention and duplication, Functional differentiation

## Abstract

**Background:**

The plant hormone auxin is widely involved in plant growth, development, and morphogenesis, and the TIR1/AFB and AUX/IAA proteins are closely linked to rapid auxin response and signal transmission. However, their evolutionary history, historical patterns of expansion and contraction, and changes in interaction relationships are still unknown.

**Results:**

Here, we analyzed the gene duplications, interactions, and expression patterns of *TIR1/AFBs* and *AUX/IAAs* to understand their underlying mechanisms of evolution. The ratios of *TIR1/AFBs* to *AUX/IAAs* range from 4:2 in *Physcomitrium patens* to 6:29 in *Arabidopsis thaliana* and 3:16 in *Fragaria vesca*. Whole-genome duplication (WGD) and tandem duplication have contributed to the expansion of the *AUX/IAA* gene family, but numerous *TIR1/AFB* gene duplicates were lost after WGD. We further analyzed the expression profiles of *TIR1/AFBs* and *AUX/IAAs* in different tissue parts of *Physcomitrium patens*, *Selaginella moellendorffii*, *Arabidopsis thaliana* and *Fragaria vesca*, and found that *TIR1/AFBs* and *AUX/IAAs* were highly expressed in all tissues in *P. patens*, *S. moellendorffii*. In *A. thaliana* and *F. vesca*, *TIR1/AFBs* maintained the same expression pattern as the ancient plants with high expression in all tissue parts, while *AUX/IAAs* appeared tissue-specific expression. In *F. vesca*, 11 AUX/IAAs interacted with TIR1/AFBs with different interaction strengths, and the functional specificity of AUX/IAAs was related to their ability to bind TIR1/AFBs, thus promoting the development of specific higher plant organs. Verification of the interactions among TIR1/AFBs and AUX/IAAs in *Marchantia polymorpha* and *F. vesca* also showed that the regulation of AUX/IAA members by TIR1/AFBs became more refined over the course of plant evolution.

**Conclusions:**

Our results indicate that specific interactions and specific gene expression patterns both contributed to the functional diversification of *TIR1/AFBs* and *AUX/IAAs*.

**Supplementary Information:**

The online version contains supplementary material available at 10.1186/s12870-023-04253-4.

## Background

Indole-3-acetic acid (IAA) is the most common and first discovered auxin in plants. Auxin is widely distributed in the plant kingdom and participates in almost all aspects of plant growth, development, and morphogenesis [1, 2]. Plants control the function of auxin mainly by coordinating auxin synthesis metabolism [3, 4], polar transport [5], and signal transduction [6]. The classical auxin signaling pathway in plants refers to the *TIR1/AFBs*-mediated auxin regulation mechanism [7, 8]. *TIR1* was the first auxin receptor protein to be identified [9, 10], and *AFBs* were subsequently discovered to also belong to this family [11–13]. The auxin receptor protein family is a subfamily of the F-Box family, whose members contain a highly conserved F-box domain and leucine repeat domain. The F-box domain of the TIR1/AFB family is an important component of the E3 ubiquitin ligase complex that participates in the degradation of AUX/IAA proteins [14, 15].

The AUX/IAA gene family is one of the key gene families involved in the rapid response to changes in auxin concentration [16, 17]. AUX/IAAs have a very short lifespan in plants, with a half-life ranging from 10 to 60 min that is determined by their domain II [18, 19]. At lower auxin levels, they bind to auxin response factor (ARF) to form a dimer that inhibits ARF and thus regulates auxin-induced gene expression. At high auxin levels, TIR1/AFB binds to AUX/IAA and degrades it through ubiquitination, thereby eliminating the inhibitory effect of AUX/IAA on ARF [20, 21]. The TIR1/AFB-AUX/IAA-ARF pathway thus describes the process of plant perception, transduction, and response to auxin signals. In addition, it was shown that the binding of auxin to TIR1/AFBs requires the co-involvement of AUX/IAAs [22]. In Arabidopsis *thaliana*, there are six TIR1/AFBs and 29 AUX/IAAs, and there may from numerous coreceptor complexes for diversification of auxin functions [23]. Also, in addition to the domain II of AUX/IAAs, different auxin concentration distributions in plants partially determine the assembly of TIR1/AFBs and AUX/IAAs [22, 23]. Thus, the SCF^TIR1/AFB^-based auxin mechanism is complex and diverse.

With the process of terrestrialization of plants, auxin has played an important role in the evolution of the original unicellular algae to the present morphologically diverse land plants. Auxin signaling genes are rare in algae and the components of signaling are severely missing [16]. For example, TIR1/AFBs were not found in all algae, while AUX/IAAs and ARFs were only found in a small fraction of algae. Until the discovery of TIR1/AFBs in bryophytes represented the emergence of the classical auxin signaling pathway [16]. The *TIR1/AFB* and *AUX/IAA* gene families have been extensively characterized in plants [24–26].However, TIR1/AFBs and AUX/IAAs exercise their functions by forming the SCF complex, and the evolutionary history of these two gene families cannot be fully understood by the analysis of a single gene family. In addition, previous studies identified auxin response proteins and their subdomains and precursors to reconstructed the origin and evolution of the auxin response system. It was shown that whole-genome duplication was the driving force behind the evolution and subfunctionalization of the *TIR1/AFB* and *AUX/IAA* gene families [27, 28]. However, the retention and loss of different branch members of *TIR1/AFBs* and *AUX/IAAs* after whole-genome duplication remains unknown.

In this study, *TIR1/AFBs* and *AUX/IAAs* were identified from 34 plants and algae, including members of the Rhodophyta, Chlorophyta, Charophyta, Bryophyta, ferns, gymnosperms, basal angiosperms, monocots, and dicots. We then reconstructed the phylogeny, synteny network, and duplication mechanisms of the *TIR1/AFBs* and *AUX/IAAs*. We compared the expression patterns of *TIR1/AFBs* and *AUX/IAAs* in different tissues of *Physcomitrium patens*, *Selaginella moellendorffii*, *A. thaliana*, and *F. vesca*, as well as their promoter *cis*-elements. According to our analysis, *Fragaria vesca* have a typical number of *TIR1/AFB* members and have remained undifferentiated during evolution. *F. vesca* was selected as a representative dicot. Bryophyte is the first taxa in which stable *TIR1/AFB* and *AUX/IAA* signaling pathways appear. Also, the smaller number of members in *Marchantia polymorpha* compared to other plants in the same group is more favorable for studying the evolutionary mode of action of ancient *TIR1/AFB* and *AUX/IAA*. In *F. vesca* and *M. polymorpha*, we explored the interactions and expression patterns between *TIR1/AFBs* and *AUX/IAAs* in detail. We found that expansion and subfunctionalization of the *TIR1/AFB* and *AUX/IAA* gene families had promoted functional specificity and the elaboration of regulatory networks.

## Results

### Genome-wide identification of ***TIR1/AFB*** and ***AUX/IAA*** gene members in plants

**Fig. 1:**
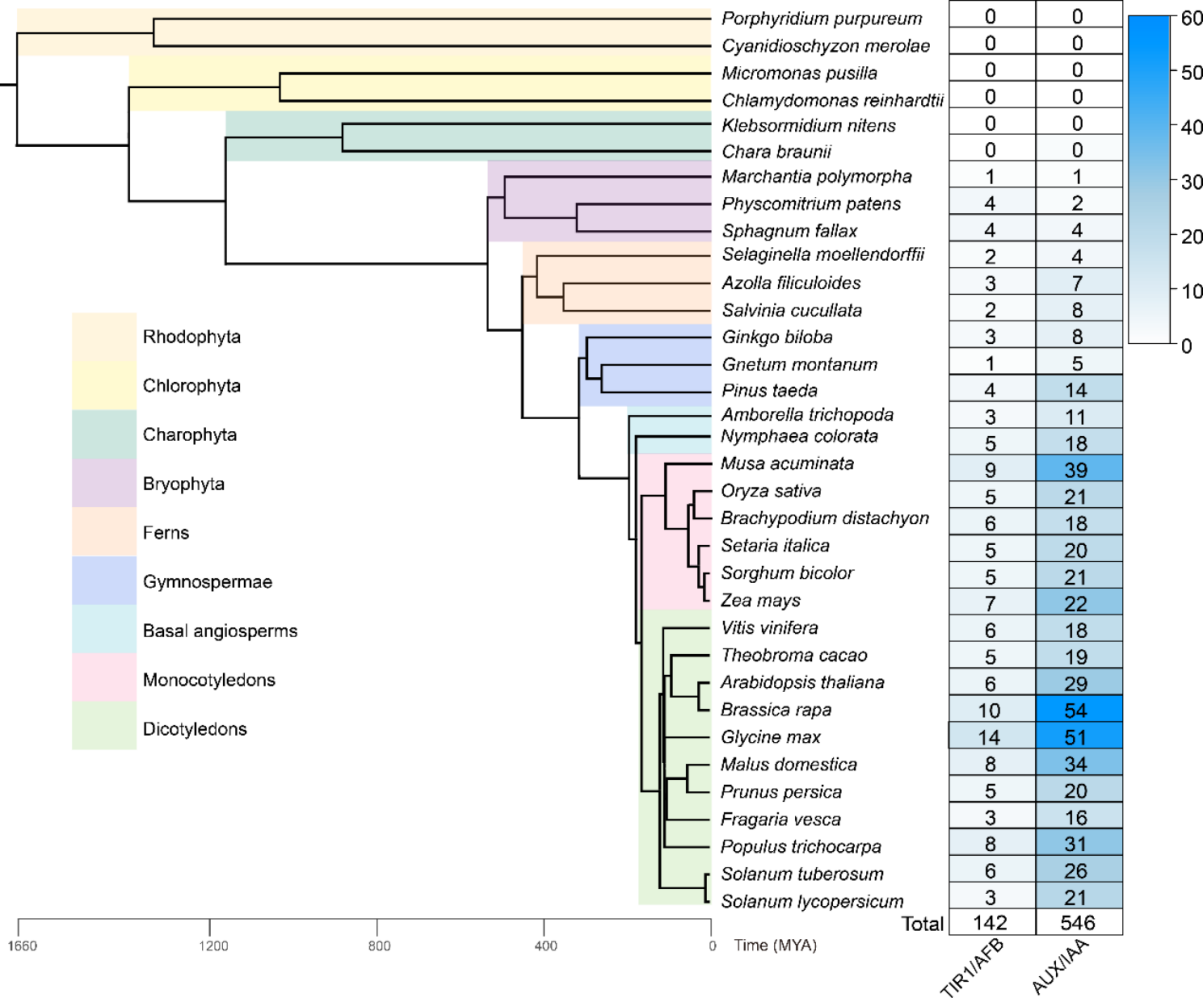
Genome-wide identification of the *TIR1/AFB* and *AUX/IAA* gene families in 34 plant species. Deep blue indicates a large number of values. Different background colors represent different plant groups

To explore the evolutionary histories of the *TIR1/AFB* and *AUX/IAA* gene families, we first used BLAST to identify the gene family members from 34 plants and algae, including Rhodophyta, Chlorophyta, Charophyta, Bryophyta, ferns, gymnosperms, basal angiosperms, monocots, and dicots. We then used HMMER to check whether the sequences contained specific domains: the Transp_inhibit (PF18791) and F-box (PF18511) domains for TIR1/ARFs and the AUX_IAA (PF02309) domain for AUX/IAAs. After filtering, there were 142 *TIR1/AFB* and 546 *AUX/IAA* candidate genes in the 34 species (Fig. 1). The results showed that *TIR1/AFB* and *AUX/IAA* first appeared in bryophytes, and the two gene families had similar numbers of members in these early plants. Over the course of evolution, there was relatively little change in the number of *TIR1/AFB* genes, but the *AUX/IAA* gene family showed marked expansion in angiosperms (Fig. 1).

**Fig. 2:**
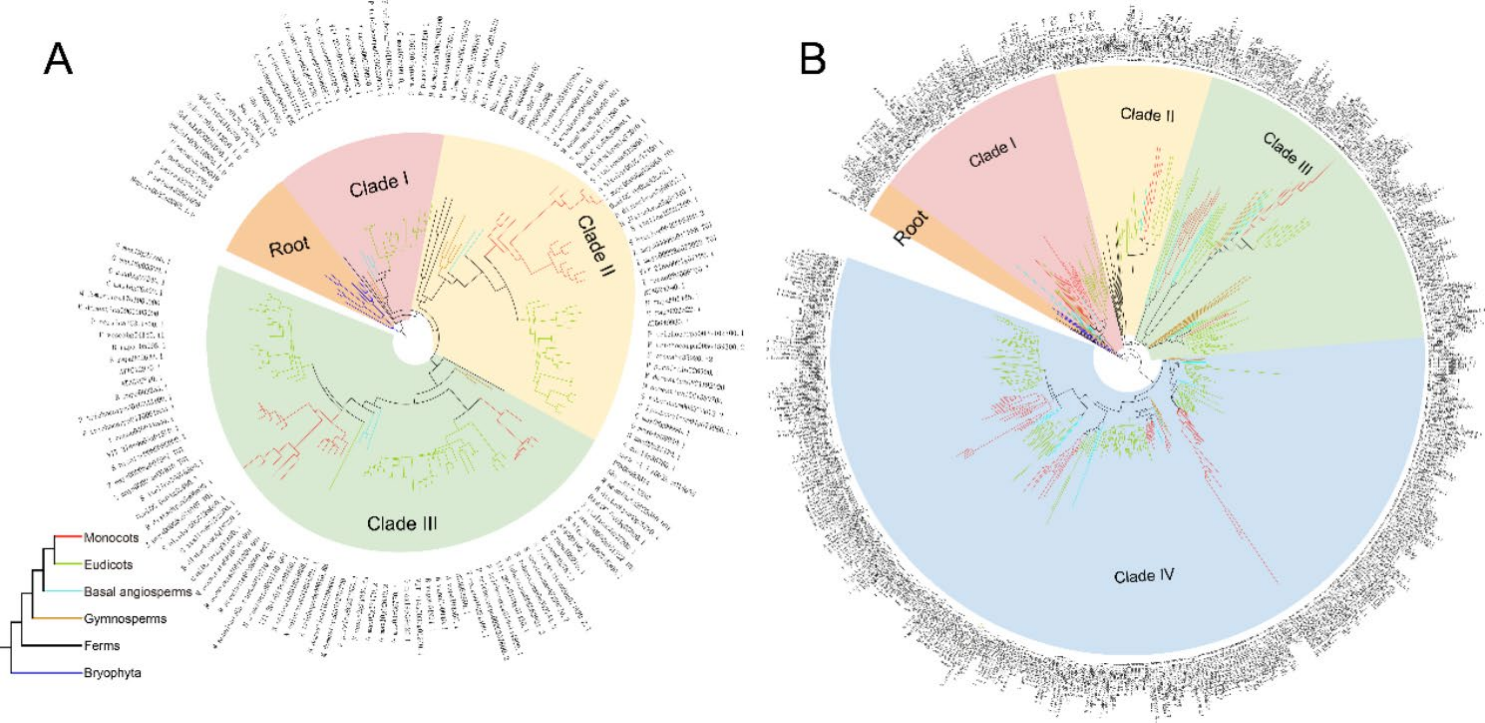
Maximum likelihood phylogenetic trees of TIR1/AFBs and AUX/IAAs. **(A)** Maximum likelihood phylogenetic tree of TIR1/AFBs. **(B)** Maximum likelihood phylogenetic tree of AUX/IAAs. The branch colors are used to distinguish Bryophyta, ferns, gymnosperms, basal angiosperms, monocots, and eudicots

To further explore their evolutionary relationships, we constructed phylogenetic trees of TIR1/AFBs and AUX/IAAs by the maximum likelihood method. The TIR1/AFB tree consisted of three main branches and could be further divided into four groups (Fig. 2A). There were no monocotyledons in clade I, and clade II contained all lineages of seed plants. *TIR1/AFB* gene families expanded in ferns, and functional differentiation of *TIR1/AFBs* occurred in monocotyledons and dicotyledons (Fig. 2A, Fig. S1). For the *AUX/IAA* gene family, there were four ancient clades and twelve groups (Fig. 2B). Functional differentiation and rapid expansion of the *AUX/IAA* gene family occurred in the gymnosperms and basal angiosperms, respectively (Fig. 2B, Fig. S1). The *AUX/IAA* gene family appeared to have a more complicated evolutionary history than the *TIR1/AFB* family.

### Phylogenomic and synteny network analyses of ***TIR1/AFBs*** and ***AUX/IAAs***

**Fig. 3:**
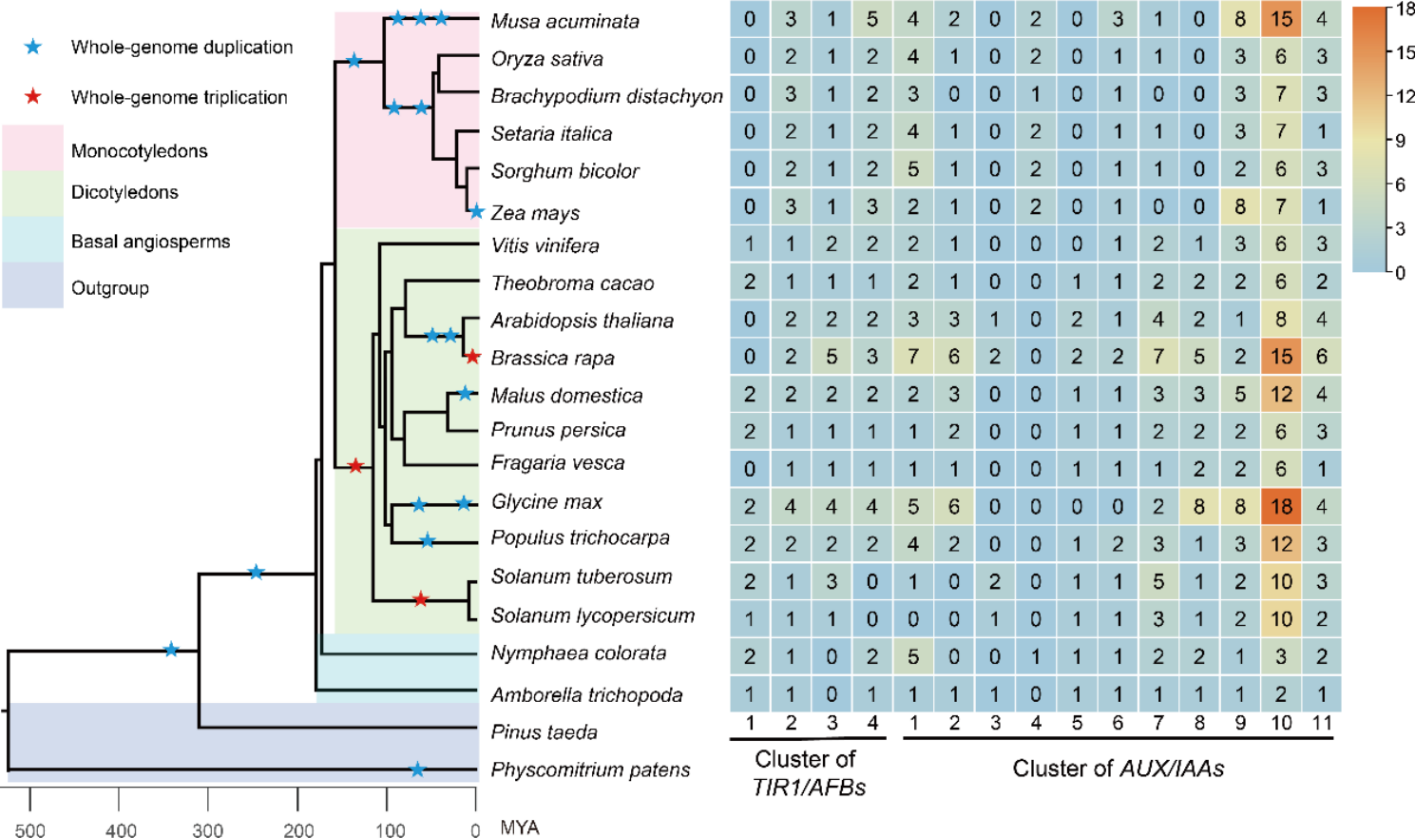
Synteny network clusters of the *TIR1/AFB* and *AUX/IAA* gene families from angiosperms. Different background colors represent different plant groups. Blue stars represent whole-genome duplication. Red stars represent whole-genome duplication

To understand the obvious differences in the evolutionary histories of plant *TIR1/AFBs* and *AUX/IAAs*, we constructed a collinearity network based on the obtained *TIR1/AFB* and *AUX/IAA* phylogenetic trees and a collinearity analysis of 19 angiosperm species. Genome-level analysis divided the *TIR1/AFB* gene family into four clusters and the *AUX/IAA* gene family into eleven clusters (Fig. 3). In the *TIR1/AFBs*, cluster 1 was specific to dicots and basal angiosperms, indicating that this group was lost in monocots. Clusters 2 and 4 were conserved in angiosperms. Cluster 3 was a new group present only in monocots and dicots (Fig. 3). In the *AUX/IAAs*, clusters 3, 5, and 8 were specific to dicots, and only cluster 4 was specific to monocots, indicating that the *AUX/IAAs* of dicots were more diverse than those of monocots. The remaining clusters contained representatives from all the angiosperms (Fig. 3).

**Fig. 4:**
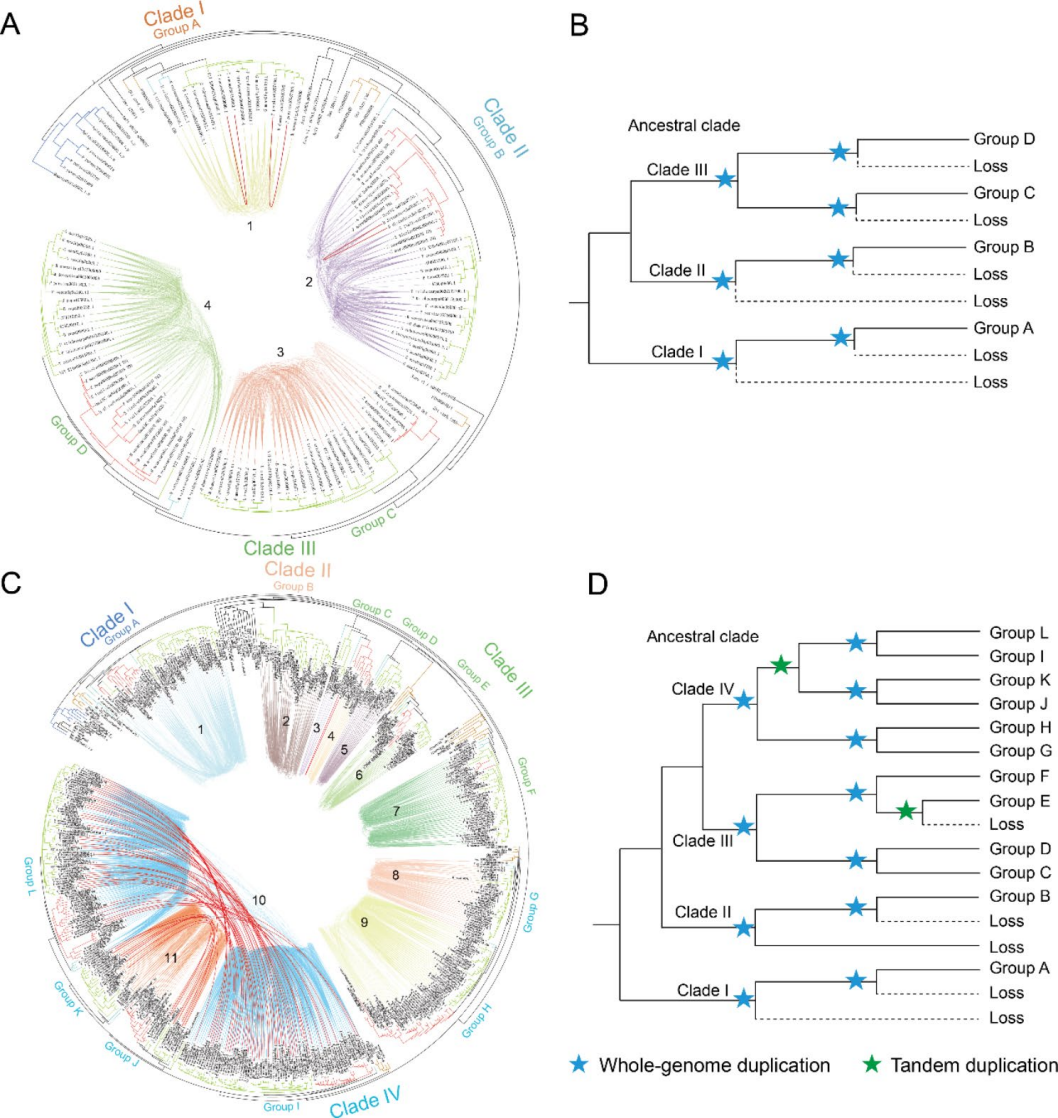
Phylogenetic profiling of the *TIR1/AFB* and *AUX/IAA* gene families. A, C: Phylogenetic and syntenic relationships of the *TIR1/AFBs* and *AUX/IAAs*. The red lines indicate tandemly duplicated gene pairs in the *TIR1/AFBs* and *AUX/IAAs*. B, D: Proposed evolutionary history of the *TIR1/AFB* and *AUX/IAA* gene families. The dashed lines indicate gene loss. Blue stars the ancient seed plant-wide and angiosperm wide genome duplication events. Green stars represent tandem duplication events of genes

Based on phylogenetic collinear network analysis, we found 747 gene pairs and 3 tandem duplications among the *TIR1/AFBs*. These tandem duplications were observed only in *Theobroma cacao*, *Prunus persica*, and *Brachypodium distachyon* (Fig. 4A). We further counted the intron numbers of the *TIR1/AFBs* and calculated the Ks (synonymous substitution) values of collinear gene pairs. Four groups of *TIR1/AFBs* shared the same intron numbers and distribution of Ks values (Fig. S2, Fig. S4A), indicating that whole-genome duplication was the primary driving force for the evolution of the *TIR1/AFB* gene family. For *AUX/IAAs*, there were 1465 gene pairs and 59 tandem duplications. One tandem duplication occurred in group C, and the rest occurred in Groups I, J, K, and L (Fig. 4C). The Ks distribution of collinear gene pairs and tandemly duplicated gene pairs showed that the *AUX/IAAs* expanded through two WGD events and one tandem duplication event, and the tandem duplication event happened between the two WGD events (Fig. S4B). Moreover, the number of introns in groups K and L decreased after the tandem duplication event (Fig. S3). The numbers of introns in group E genes were also significantly reduced (Fig. S3). We therefore speculated that group E may have experienced tandem duplication independently after the second WGD event and the original branch was lost in subsequent evolution.

According to these results, we constructed a possible evolutionary history of *TIR1/AFBs* and *AUX/IAAs*. In the *TIR1/AFB* family, we hypothesized that there were already three *TIR1/AFB* members in the ancestors of seed plants, and the *TIR1/AFB* family expanded further through two WGD events (Fig. 4B). In the *AUX/IAA* family, there were four members in the common ancestor of seed plants. The four ancestral clades expanded through the two WGD events and two additional tandem duplication events in seed plants (Fig. 4D). Therefore, the evolution and expansion of the *AUX/IAA* gene family were driven by tandem duplication and WGD.

### Functionally specialized ***AUX/IAAs*** show enhanced interaction ability with ***TIR1/AFBs***

To investigate the functional differentiation of *TIR1/AFB* and *AUX/IAA* members in plants, we constructed heat maps showing their expression in different tissues of *P. patens*, *S. moellendorffii*, *A. thaliana*, and *F. vesca*. All *TIR1/AFBs* were highly expressed in different tissues of the four species (Fig. S5-S8), demonstrating that *TIR1/AFB* members were functionally conserved and extensively involved in plant morphogenesis. All *AUX/IAAs* were highly expressed in different tissues of *P. patens* and *S. moellendorffii* (Fig. S5, S6). These results suggest that when the *TIR1/AFB*-*AUX/IAA* signaling pathway appeared in ancient plants, it was likely to have been involved in all aspects of plant development. Some groups of *AUX/IAAs* maintained the same expression patterns in *A. thaliana* and *F. vesca* as in *P. patens* and *S. moellendorffii* (groups A, F, G, H, I, and L in *A. thaliana* and groups A, D, G, and H in *F. vesca*) (Fig. S5–S8). However, *AUX/IAAs* in the remainder of the groups appeared to have begun functional differentiation and were highly expressed in only one of the vegetative or reproductive organs; these included groups B and D of *A. thaliana*, focused on seed development, and group F of *F. vesca*, also focused on seed development (Fig. S7, S8). In addition, the functional differentiation of *AUX/IAAs* was more clearly in *F. vesca* than in *A. thaliana*.

**Fig. 5:**
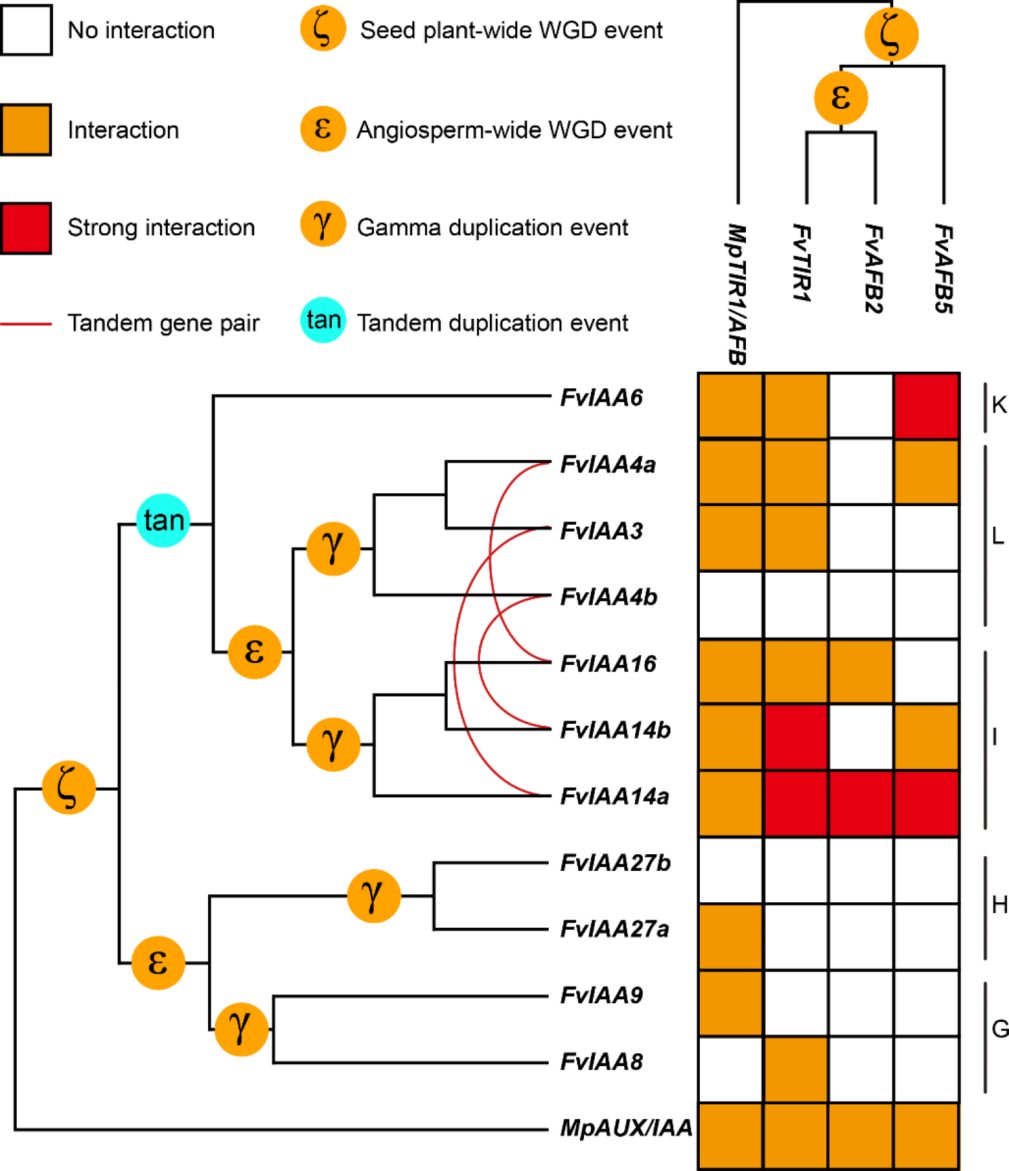
Interaction patterns of *TIR1/AFBs* and *AUX/IAAs* from *F. vesca* and *M. polymorpha*. The colors on the interaction heatmap represent the intensity of interactions between different genes. Red, Strong interaction; orange, interaction; white, no interaction. The red lines represent tandem gene pair. ζ, seed plant-wide WGD event; ε, angiosperm-wide WGD event; γ, gamma duplication event; tan, tandem dupliacation event

In addition, clade IV appeared to have experienced the most complex duplication history (Fig. 4D). Therefore, we constructed the evolutionary trajectories of *TIR1/AFBs* and *AUX/IAAs* in *F. vesca* and *M. polymorpha* and verified their interaction patterns (Fig. 5, Fig. S12). The results showed that FvTIR1, FvAFB2, and FvAFB5 could interact with 7, 2, and 4 FvAUX/IAA members, respectively (Fig. 5, Fig. S10). Furthermore, we found that FvIAA14a strongly interacted with all FvTIR1/AFBs, and FvIAA14b and FvIAA6 strongly interacted with FvTIR1 and FvAFB5, respectively (Fig. 5, Fig. S11). Interestingly, AUX/IAAs in groups G and H showed little interaction with the TIR1/AFBs, even though they shared a common ancestor with groups I, J, K, and L (Fig. 5, Fig. S11). AUX/IAAs in groups G and H appeared to be involved in the development of all tissues. However, AUX/IAAs in groups I, J, K, and L (except *FvIAA16*) showed tissue-specific expression (Fig. S8). Therefore, we speculated that the enhanced binding ability of AUX/IAAs for TIR1/AFBs may have promoted the development of functional specificity. In clade IV, the functional specificity of AUX/IAAs in reproductive organs was related to their ability to interact with TIR1/AFBs. The binding ability of FvIAA3 and FvIAA4b with FvTIR1/AFBs was weakened after tandem duplication events, and their expression in reproductive organs also disappeared (Fig. 5, Fig. S8). In addition, we comprehensively analyzed the promoter of *TIR1/AFB* and *AUX/IAA* gene families in *P. patens*, *S. moellendorfii*, *A. thaliana*, and *F. vesca*. The results showed that a large number of hormone responsive elements (Abscisic acid, Gibberellin, Methyl jasmonate, Salicylic acid., Auxin) present in the promoter. Furthermore, *cis*-elements related to growth and development are also specifically present in different *TIR1/AFB* and *AUX/IAA* member promoters (Fig. S9). These results suggested that changes in interaction modes after duplication events and regulatory elements of the promoter may have helped to promote the specific development of higher plant organs, at least to some extent.

To further investigate these results, we confirmed the interaction patterns of TIR1/AFBs and AUX/IAAs between *F. vesca* and *M. polymorpha*. As shown in Fig. 5, MpTIR1/AFB interacted with eight FvAUX/IAAs, and MpAUX/IAA could interact with all TIR1/AFBs of F. vesca and M. polymorpha. This result showed that ancient TIR1/AFB and AUX/IAA had broad binding capacity but lacked the ability to be precisely regulated. Compared with *M. polymorpha*, strawberry displayed a more elaborate regulatory network formed by the subfunctionalization of genes that arose through the expansion of the *TIR1/AFB* and *AUX/IAA* gene families.

## Discussion

The terrestrialization of aquatic plants was an important event in plant evolution. However, the time at which the auxin signal appeared is still unknown [29]*TIR1/AFBs* and *AUX/IAAs* have been found in a large number of land plants, ranging from lower plants such as mosses to higher plants. Thus, the *TIR1/AFBs*-*AUX/IAAs* signaling pathway is conserved in land plants [24, 30–32]. In this study, *TIR1/AFBs* was identified in many mosses and ferns, but no homologous genes were found in the surveyed algae. Although auxin signals and *AUX/IAA*s had already emerged in algae [16, 33, 34], the absence of classical auxin signaling pathway elements indicated that this pathway first appeared in bryophytes. It has been suggested that this phenomenon may have been caused by a massive loss of ubiquitinated components in algae [34].

Whole-genome duplication doubles the entire genome of the plant and is thus a major mechanism of gene family expansion. In addition, tandem duplication also promotes the expansion of gene families [35, 36]. However, a large number of gene copies are lost during the evolutionary process [37, 38], and the retained genes promote better adaptation of plant growth and development [39–42]. *TIR1/AFBs* have experienced two WGDs in land plants, but the number of *TIR1/AFBs* has not expanded substantially, and they are divided into only three different types [27]. The *AUX/IAAs* not only experienced the same WGD events as the *TIR1/AFBs* but also experienced two large-scale tandem duplication events in angiosperms [27]. Although the evolutionary patterns of *TIR1/AFBs* and *AUX/IAAs* have been explored previously, this paper describes their retention and loss after duplication event in more detail. Also, two tandem duplication events that promote *AUX/IAAs* expansion were identified. Large numbers of *AUX/IAAs* were retained after duplication. Thus, the number of *AUX/IAAs* is greater than the number of *TIR1/AFBs* in seed plants. In Bryophyta, the ratio of *TIR1/AFBs* and *AUX/IAAs* was 1 or more. However, the rapid expansion of *AUX/IAAs* altered the balance between *TIR1/AFBs* and *AUX/IAAs*.

Gene subfunctionalization is a key mechanism for duplicate gene retention, and the main phenomenon of subfunctionalization is a temporal and/or spatial differentiation of gene expression [43]. The *TIR1/AFBs* were all highly expressed in multiple tissues of *P. patens*, *S. moellendorffii*, *A. thaliana*, and *F. vesca*. Thus, a large number of *TIR1/AFB* duplicates were not retained after WGDs. By contrast, from lower land plants to higher plants, duplicated *AUX/IAA* genes were retained because of gene expression divergence. The functional differentiation of *AUX/IAAs* was most obvious in *F. vesca*. Thus, subfunctionalization and duplicate retention explained the marked expansion of *AUX/IAAs* in higher plants.

The expansion of *AUX/IAAs* would result in *AUX/IAAs* competing with one another for binding to *TIR1/AFBs*. Therefore, the interaction relationships between *TIR1/AFBs* and *AUX/IAAs* might be retained, lost, or gained over evolutionary history. We focused on clade IV, which was the most complex branch in *AUX/IAA* evolutionary history, and we analyzed the interaction relationships among *AUX/IAAs* and *TIR1/AFBs* of *F. vesca*. We found that the binding ability between *F. vesca TIR1/AFBs* and *AUX/IAAs* was closely related to the functional specificity of the *F. vesca AUX/IAAs. AUX/IAAs* with stronger functional specificity were more stringently regulated by *TIR1/AFBs*. This was also demonstrated by the binding relationships among *TIR1/AFBs* and *AUX/IAAs* from *M. polymorpha* and *F. vesca*. In addition, the analysis of the strong and weak binding ability of *TIR1/AFBs* and *AUX/IAAs* had been similarly studied in previous studies. In *A. thaliana*, *AtIAA7* had the strongest binding capacity to *AtTIR1/AFBs*. It can bind to *AtTIR1* and *AtAFB2* in the absence of auxin and to all *AtTIR1/AFBs* in the presence of trace amounts of auxin. In contrast, its tandem duplication gene *AtIAA3* only bound to *AtAFB1* in the presence of trace auxin and fails to bind to *AtAFB5* at high auxin concentrations [22]. In addition, the functions of *AtIAA7* and *AtIAA3* in plant development and root growth were similar [44–55], and *AtIAA7* was also able to regulate flowering time in *A. thaliana* [56, 57] while the function of *AtIAA3* in flower development had not been reported yet. This was identical to the expression profiles of *AtIAA7* and *AtIAA3* in different tissue parts of *A. thaliana*. The expression of IAA3 was significantly lower in reproductive organs than in nutritional organs. Thus, we hypothesize that the conserved functions of Arabidopsis and strawberry in Vegetative tissue was its origin from their ancestors, but as plants evolved, organ specificity led to the emergence of more refined regulation. This phenomenon could strongly promote accurate regulation of the development of diverse organs in higher plants by auxin.

## Conclusions

In this study, *TIR1/AFBs* and *AUX/IAAs* were identified from 34 plants and algae, including members of the Rhodophyta, Chlorophyta, Charophyta, Bryophyta, ferns, gymnosperms, basal angiosperms, monocots, and dicots. We found that members of *TIR1/AFBs* and *AUX/IAAs* were incomplete in basal plants. Their appearance was associated with the terrestrialization of plants and appears intact for the first time in bryophytes. Meanwhile, whole genome duplication (WGD) and tandem duplication promoted the expansion of the *AUX/IAA* gene family, but many *TIR1/AFB* gene duplications were lost after WGD. In the expression profiles of different plants, *TIR1/AFBs* were found to be highly expressed in all tissue sites, whereas *AUX/IAAs* were highly expressed in all tissue sites in mosses and ferns, but tissue-specific expression was observed in higher plants. Also, further binding experiments showed that the functional specificity of AUX/IAAs was related to their ability to bind TIR1/AFBs. Our results indicate that specific interactions and specific gene expression patterns both contributed to the functional diversification of *TIR1/AFBs* and *AUX/IAAs*.

Methods.

Identification of *TIR1/AFBs* and *AUX/IAAs*.

Thirty-four plant genome sequences were downloaded from Phytozome v13 (https://phytozome-next.jgi.doe.gov/) and other websites (Table S1). The 6 TIR1/AFBs and 29 AUX/IAAs of *A. thaliana* were used as query sequences, and BLASTP v2.10.0 [58] was performed against the 33 other plant proteins with E-value < 1E − 10. We further confirmed the BLAST hits using HMMER v3.3.2 [59]. TIR1/AFB proteins were required to contain the Transp_inhibit (PF18791) and F-box (PF18511) domains, and the AUX/IAA proteins were required to contain the AUX_IAA (PF02309) domain. Finally, we constructed evolutionary trees using FastTree v2.10.0 [60] and manually filtered extremely long branches.

Reconstructed phylogenies of TIR1/AFBs and AUX/IAAs.

The protein sequences of the TIR1/AFBs and AUX/IAAs were aligned using MUSCLE v3.8.1551 [61], and phylogenetic trees were reconstructed using RAxML v8.2.12 [62] with the GTRGAMMA model and 100 bootstrap replicates. Finally, we visualized the phylogenetic trees using the ITOL website [63].

Synteny, duplication, and gene-pair Ks analysis.

Collinearity networks were identified using the python version of MCScan (JCVI v1.1.7) [64] by comparing coding sequences to coding sequences. Then, the DupGen_finder pipeline [65] was performed to investigate potential duplication events. The Ks values of all gene pairs were calculated using KaKs_Calculator v2.0 [66].

Gene expression analysis.

The expression profile of *P. patens* was downloaded from the Physcomitrium eFP Browser (http://bar.utoronto.ca/efp_physcomitrella/cgi-bin/efpWeb.cgi). The expression profile of *S. moellendorffii* was downloaded from the Selaginella eFP Browser (http://bar.utoronto.ca/efp_selaginella/cgi-bin/efpWeb.cgi), and RNA sequencing (RNA-seq) data from spores were downloaded from NCBI (PRJNA326972). We obtained expression data for *A. thaliana* from the Arabidopsis eFP Browser (http://bar.utoronto.ca/efp/cgi-bin/efpWeb.cgi). Expression data for *F. vesca* were downloaded from the Strawberry eFP Browser (http://bar.utoronto.ca/efp_strawberry/cgi-bin/efpWeb.cgi), and RNA-seq data from old leaves, roots and shoots of *F. vesca* were downloaded from NCBI (PRJNA695578). We mapped all RNAseq data to the genome using HISAT2 v2.2.1 [67] and SAMtools v1.7.1 [68]. The genome version used was listed in Table S1. Then, the FPKM (Fragments Per Kilobase of exon model per Million mapped fragments) value were generated with Subread v2.0.1 [69] and Trinity v2.13.2 [70]. Finally, we visualized the expression data using TBtools [71].

Promoter *cis*-element analysis.

The promoter (2000 bp upstream) of *TIR1/AFB* and *AUX/IAA* gene families in *P. patens*, *S. moellendorfii*, *A. thaliana*, and *F. vesca* were extracted. Then, the *cis*-elements were predicted by online website PlantCARE (http://bioinformatics.psb.ugent.be/webtools/plantcare/html/). Finally, we visualized the *cis*-elements by ITOL website [63].

RNA extraction, complementary DNA preparation, and vector construction.

Total RNA was extracted from *Fragaria vesca* ‘Hawaii-4’ seedlings and *M. polymorpha* mature plant using a Plant Total RNA Isolation Kit (FOREGENE, Chengdu, China). The plant materials used in this experiment were obtained from the Fruit Tree Phylogenetic Laboratory of Nanjing Agricultural University. According to the instructions of the PrimeScript RT reagent kit (TaKaRa, Beijing, China), we synthesized the complementary DNA for gene cloning. Then, we cloned full-length *TIR1/AFBs* and *AUX/IAAs* using PrimeSTAR HS DNA Polymerase (TaKaRa, Beijing, China). Finally, the full-length genes were ligated into the pGADT7 and pGBKT7 vectors with the GenRec Assembly Master Mix Kit (GENERAL BIOL, Anhui, China). The primers used in this study are listed in Table S2 and Information of all amplified genes was listed in Table S3.

Yeast two-hybrid interaction assays.

Yeast two-hybrid interaction assays were performed to investigate the interactions of *TIR1/AFBs* with *AUX/IAAs*. Twelve *AUX/IAA* genes were ligated into the pGADT7 vector as prey, and four *TIR1/AFB* genes were ligated into the pGBKT7 vector as bait. Then, all constructed vectors and control vectors were transformed into the yeast strain Y2H Gold by the modified lithium acetate method. Finally, the transformed yeasts were cultured on SD-Leu-Trp medium, SD-Leu-Trp-His + X-α-Gal medium, and SD-Leu-Trp-His-Ade + X-α-Gal medium. For interactions, we classified them as interaction and strong interaction. Interactions: yeast could grow in SD-Leu-Trp-His + X-α-Gal medium and turn blue; strong interaction: yeast could grow in SD-Leu-Trp-His-Ade + X-α-Gal medium and turn blue. In addition, the interaction of TIR1/AFBs and AUX/IAAs may be influenced by auxin. Therefore, we added different concentrations of indole-3-acetic acid (0, 0.1, 1, 10 µM) to the SD-Leu-Trp-His + X-α-Gal medium, and SD-Leu-Trp-His-Ade + X-α-Gal medium.

## Electronic supplementary material

Below is the link to the electronic supplementary material.

Supplementary Material 1

## Data Availability

The datasets analysed during the current study are available in the Physcomitrium eFP Browser (http://bar.utoronto.ca/efp_physcomitrella/cgi-bin/efpWeb.cgi), the Selaginella eFP Browser (http://bar.utoronto.ca/efp_selaginella/cgi-bin/efpWeb.cgi), the Arabidopsis eFP Browser (http://bar.utoronto.ca/efp/cgi-bin/efpWeb.cgi) and the Strawberry eFP Browser (http://bar.utoronto.ca/efp_strawberry/cgi-bin/efpWeb.cgi) repository. The raw RNA-seq data used in this study can obtained at NCBI website (PRJNA326972 and PRJNA695578).

## Declarations

### Experimental research on plants

Experimental research was carried out with relevant institutional, national, and international guidelines and legislation.

## References

[1] Kepinski S, Leyser O (2005). Plant development: auxin in loops. CURR BIOL.

[2] Woodward AW, Bonnie B (2005). Auxin: Regulation, Action, and Interaction. Ann Bot.

[3] Zhao Y (2010). Auxin Biosynthesis and its role in Plant Development. ANNU REV PLANT BIOL.

[4] Zhao Y (2018). Essential roles of local Auxin Biosynthesis in Plant Development and in adaptation to environmental changes. ANNU REV PLANT BIOL.

[5] Petrasek J, Friml J (2009). Auxin transport routes in plant development. DEVELOPMENT.

[6] Mockaitis E (2008). Auxin Receptors and Plant Development: a New Signaling paradigm. ANNU REV CELL DEV BIOL.

[7] Leyser O (2017). Auxin Signaling. PLANT PHYSIOL.

[8] Martin K, Richard N (2019). Non-canonical auxin signalling: fast and curious. J EXP BOT.

[9] Dharmasiri N, Dharmasiri S, Estelle M (2005). The F-box protein TIR1 is an auxin receptor. Nature.

[10] Kepinski S, Leyser O (2005). The Arabidopsis F-box protein TIR1 is an auxin receptor. Nature.

[11] Dharmasiri N, Dharmasiri S, Weijers D, Lechner E, Yamada M, Hobbie L, Ehrismann JS, Jürgens G, Estelle M (2005). Plant development is regulated by a family of auxin receptor F box proteins. DEV CELL.

[12] Hu Z, Ke Eli MA, Piisil M, Li J, Survila M, Heino P, Brader G, Palva ET, Li J (2012). F-box protein AFB4 plays a crucial role in plant growth, development and innate immunity. CELL RES.

[13] Prigge MJ, Greenham K, Zhang Y, Santner A, Castillejo C, Mutka AM, O’Malley RC, Ecker JR, Kunkel BN, Estelle M. The Arabidopsis Auxin Receptor F-Box Proteins AFB4 and AFB5 Are Required for Response to the Synthetic Auxin Picloram. *G3 Genesgenetics* 2016, 6(5):1383–1390. 10.1534/g3.115.025585 PMC4856089 26976444

[14] Ruegger M, Dewey E, Gray WM, Hobbie L, Turner J, Estelle M (1998). The TIR1 protein of Arabidopsis functions in auxin response and is related to human SKP2 and yeastGrr1p. Genes Dev.

[15] Ruegger M, Dewey E, Hobbie L, Brown D, Bernasconi P, Turner J, Muday G, Estelle M (1997). Reduced naphthylphthalamic acid binding in the tir3 mutant of Arabidopsis is associated with a reduction in polar auxin transport and diverse morphological defects. PLANT CELL.

[16] Jiao C, Sørensen I, Sun X, Sun H, Behar H, Alseekh S, Philippe G, Palacio Lopez K, Sun L, Reed R (2020). The Penium margaritaceum genome: Hallmarks of the Origins of Land plants. Cell.

[17] Paponov IA, Paponov M, Teale W, Menges M, Chakrabortee S, Murray JAH, Palme K (2008). Comprehensive transcriptome analysis of auxin responses in Arabidopsis. MOL PLANT.

[18] Oeller PW, Keller JA, Parks JE, Silbert JE, Theologis A (1993). Structural characterization of the early indoleacetic acid-inducible genes, PS-IAA4/5 and PS-IAA6, of pea (Pisum sativum L). J MOL BIOL.

[19] Yasushi SM, Tatsuo K (2014). Auxin sensitivities of all Arabidopsis Aux/IAAs for degradation in the presence of every TIR1/AFB. Plant Cell Physiol.

[20] Dharmasiri N, Dharmasiri S, Jones AM, Estelle M (2003). Auxin Action in a cell-free system. CURR BIOL.

[21] Li S, Xie Z, Hu C, Zhang J (2016). A review of Auxin Response factors (ARFs) in plants. FRONT PLANT SCI.

[22] Calderón Villalobos LIA, Lee S, De Oliveira C, Ivetac A, Brandt W, Armitage L, Sheard LB, Tan X, Parry G, Mao H (2012). A combinatorial TIR1/AFB–Aux/IAA co-receptor system for differential sensing of auxin. NAT CHEM BIOL.

[23] Salehin M, Bagchi R, Estelle M (2015). SCFTIR1/AFB-Based Auxin Perception: mechanism and role in Plant Growth and Development. Plant Cell.

[24] Wu W, Liu Y, Wang Y, Li H, Liu J, Tan J, He J, Bai J, Ma H (2017). Evolution analysis of the Aux/IAA gene family in plants shows dual Origins and Variable Nuclear localization signals. INT J MOL SCI.

[25] Shu W, Liu Y, Guo Y, Zhou H, Zhang J, Zhao S, Lu M. A Populus TIR1 gene family survey reveals differential expression patterns and responses to 1-naphthaleneacetic acid and stress treatments. FRONT PLANT SCI 2015, 6. 10.3389/fpls.2015.00719 PMC4585115 26442033

[26] Cai Z, Zeng D, Liao J, Cheng C, Sahito ZA, Xiang M, Fu M, Chen Y, Wang D. Genome-Wide Analysis of Auxin Receptor Family Genes in Brassica juncea var. tumida. In: *Genes*, vol. 10; 2019. 10.3390/genes10020165 PMC6410323 30791673

[27] Mutte SK, Kato H, Rothfels C, Melkonian M, Wong GK, Weijers D, Yu H (2018). Origin and evolution of the nuclear auxin response system. ELIFE.

[28] Das S, Weijers D, Borst JW (2021). Auxin Response by the numbers. TRENDS PLANT SCI.

[29] Smet ID, Beeckman T (2011). Asymmetric cell division in land plants and algae: the driving force for differentiation. NAT REV MOL CELL BIO.

[30] Lau S, Shao N, Bock R, Jürgens G, Smet ID (2009). Auxin signaling in algal lineages: fact or myth?. TRENDS PLANT SCI.

[31] Paponov IA, Teale W, Lang D, Paponov M, Reski R, Rensing SA, Palme K (2009). The evolution of nuclear auxin signalling. BMC EVOL BIOL.

[32] Parry G, Calderon-Villalobos LI, Prigge M, Peret B, Dharmasiri S, Ltoh H, Lechner E, Gray WM, Bennett M, Estelle M (2009). Complex regulation of the TIR1/AFB family of auxin receptors. Proc Natl Acad Sci U S A.

[33] Ohtak K, Hor K, Kanno Y, Seo M, Oht H (2017). Primitive Auxin response without TIR1 and Aux/IAA in the Charophyte Alga Klebsormidium nitens. PLANT PHYSIOL.

[34] Hori K, Maruyama F, Fujisawa T, Togashi T, Yamamoto N, Seo M, Sato S, Yamada T, Mori H, Tajima N (2014). Klebsormidium flaccidum genome reveals primary factors for plant terrestrial adaptation. NAT COMMUN.

[35] Freeling M (2009). Bias in Plant Gene Content following different sorts of duplication: Tandem, Whole-Genome, Segmental, or by Transposition. ANNU REV PLANT BIOL.

[36] Panchy N, Lehti-Shiu MD, Shiu SH (2016). Evolution of gene duplication in plants. PLANT PHYSIOL.

[37] Conant GC, Birchler JA, Pires JC (2014). Dosage, duplication, and diploidization: clarifying the interplay of multiple models for duplicate gene evolution over time. CURR OPIN PLANT BIOL.

[38] Lynch M, Conery JS (2000). The Evolutionary Fate and Consequences of Duplicate genes. Science.

[39] Liu S, Liu Y, Yang X, Tong C, Edwards D, Parkin IAP, Zhao M, Ma J, Yu J, Huang S (2014). The Brassica oleracea genome reveals the asymmetrical evolution of polyploid genomes. NAT COMMUN.

[40] Maere S, Bodt SD, Raes J, Casneuf T, Montagu MV, Kuiper M, Peer Y (2005). Modeling gene and genome duplications in eukaryotes. P NATL ACAD SCI USA.

[41] Schnable JC, Springer NM, Freeling M (2011). Differentiation of the maize subgenomes by genome dominance and both ancient and ongoing gene loss. P NATL ACAD SCI USA.

[42] Wu Y, Zhu Z, Ma L, Chen M (2008). The Preferential Retention of Starch synthesis genes reveals the impact of whole-genome duplication on Grass Evolution. Mol Biology Evol.

[43] Orce AF, Lynch M, Pickett FB, Amores A, Postlethwait J. Preservation of Duplicate Genes by Complementary, Degenerative Mutations. *GENETICS* 1999, 151(4):1531–1545. 10.1093/genetics/151.4.1531 PMC1460548 10101175

[44] Belin M, Hauserova L-M. Abscisic Acid Represses Growth of the Arabidopsis Embryonic Axis after Germination by Enhancing Auxin Signaling. PLANT CELL 2009. 10.1105/tpc.109.067702 PMC2751952 19666738

[45] Dandan C, Terese R, Shoucheng C, C LM, Anne LR, Gang-Ping X. Drought-Up-Regulated TaNAC69-1 is a Transcriptional Repressor of TaSHY2 and TaIAA7, and Enhances Root Length and Biomass in Wheat. Plant Cell Physiol 2016, 57(10). 10.1093/pcp/pcw126 27440550

[46] Goh T, Kasahara H, Mimura T, Fukaki KH (2012). Multiple AUX/IAA—ARF modules regulate lateral root formation: the role of Arabidopsis SHY2/IAA3-mediated auxin signalling. Philosophical Trans Biol Sci.

[47] Kim BC, Soh MC, Kang BJ, Furuya M, Nam HG (2010). Two dominant photomorphogenic mutations of Arabidopsis thaliana identified as suppressor mutations of hy2. PLANT J.

[48] Koren D, Resnick N, Gati EM, Belausov E, Koltai H. Strigolactone signaling in the endodermis is sufficient to restore root responses and involves SHORT HYPOCOTYL 2 (SHY2) activity. NEW PHYTOL 2013, 198(3). 10.1111/nph.12189 23425316

[49] Li T, Kang X, Lei W, Yao X, Zou L, Zhang D, Lin H (2020). SHY2 as a node in the regulation of root meristem development by auxin, brassinosteroids, and cytokinin. J INTEGR PLANT BIOL.

[50] Lujun Y, Xiaodong C, Qi C, Dongqing W, Xiang-Yang H, Ai-Qun J (2022). Diketopiperazine modulates Arabidopsis thaliana Root System Architecture by promoting interactions of auxin receptor TIR1 and IAA7/17 proteins. PLANT CELL PHYSIOL.

[51] Raffaele DI, Francisco Scaglia L, Emanuele S, Eva C, Renze H, Paolo C, Sabrina S. Cytokinins determine Arabidopsis root-meristem size by controlling cell differentiation. Curr biology: CB 2007, 17(8). 10.1016/j.cub.2007.02.047 17363254

[52] Reed JW, Elumalai RP, Chory J. Suppressors of an Arabidopsis thaliana phyB mutation identify genes that control light signaling and hypocotyl elongation. *GENETICS* 1998, 148(3):1295. 10.1093/genetics/148.3.1295 PMC1460030 9539443

[53] Tian Q, Reed JW (1999). Control of auxin-regulated root development by the Arabidopsis thaliana SHY2/IAA3 gene. DEVELOPMENT.

[54] Timpte C, Wilson A, Estelle M (1995). The Axr2-1 mutation of Arabidopsis Thaliana is a gain-of-function mutation that disrupts an early step in Auxin Response. Genetics.

[55] Wilson AK, Pickett FB, Turner JC, Estelle M (1990). A dominant mutation inArabidopsis confers resistance to auxin, ethylene and abscisic acid. Mol Gen Genet MGG.

[56] Atsuko S, Shu S, Jun M, Kotaro TY. Negative phototropism is seen in Arabidopsis inflorescences when auxin signaling is reduced to a minimal level by an Aux/IAA dominant mutation, axr2. Plant Signal Behav 2015, 10(3). 10.4161/15592324.2014.990838 PMC4622695 25738325

[57] Mai YX, Wang L, Yang HQ (2011). A gain-of-function mutation in IAA7/AXR2 confers late flowering under short-day light in Arabidopsis. J INTEGR PLANT BIOL.

[58] Altschul SF (2012). Basic local alignment search tool (BLAST). J MOL BIOL.

[59] Johnson LS, Ed Dy SR, Portugaly E (2010). Hidden Markov model speed heuristic and iterative HMM search procedure. BMC Bioinformatics.

[60] Price MN, De Hal PS, Arkin AP (2010). FastTree 2 – approximately maximum-likelihood trees for large alignments. PLoS ONE.

[61] Edgar RC (2004). MUSCLE: multiple sequence alignment with high accuracy and high throughput. NUCLEIC ACIDS RES.

[62] Alexandros S (2014). RAxML version 8: a tool for phylogenetic analysis and post-analysis of large phylogenies. Bioinformatics.

[63] Ivica L, Peer B. Interactive Tree Of Life v2: online annotation and display of phylogenetic trees made easy. NUCLEIC ACIDS RES 2011, 39(Web Server issue):W475–8. 10.1093/nar/gkr201 PMC3125724 21470960

[64] Tang H, Bowers JE, Wang X, Ming R, Alam M, Paterson AH (2008). Synteny and collinearity in Plant Genomes. Science.

[65] Qiao X, Li Q, Yin H, Qi K, Li L, Wang R, Zhang S, Paterson AH (2019). Gene duplication and evolution in recurring polyploidization–diploidization cycles in plants. GENOME BIOL.

[66] Wang D, Zhang Y, Zhang Z, Jiang Z, Jun Y (2010). KaKs_Calculator 2.0: a Toolkit incorporating Gamma-Series methods and sliding window strategies. GENOM PROTEOM BIOINF.

[67] Kim D, Langmead B, Salzberg SL (2015). HISAT: A fast spliced aligner with low memory requirements. NAT METHODS.

[68] Li H, Handsaker B, Wysoker A, Fennell T, Ruan J, Homer N, Marth G, Abecasis G, Durbin R (2009). The sequence Alignment/Map format and SAMtools. Bioinformatics.

[69] Yang L, Gordon K, Smyth, Wei S (2013). The subread aligner: fast, accurate and scalable read mapping by seed-and-vote. NUCLEIC ACIDS RES.

[70] Grabherr MG, Haas BJ, Yassour M, Levin JZ, Thompson DA, Amit I, Adiconis X, Fan L, Raychowdhury R, Qiandong Z (2013). Trinity: reconstructing a full-length transcriptome without a genome from RNA-Seq data. NAT BIOTECHNOL.

[71] Chen C, Chen H, Zhang Y, Thomas HR, Xia R (2020). TBtools: an integrative Toolkit developed for interactive analyses of big Biological Data. MOL PLANT.

