# Supplementary material for: Different evolutionary patterns of TIR1/AFBs and AUX/IAAs and their implications for the morphogenesis of land plants
Source: BMC Plant Biol. 2023 May 19;23:265. doi: 10.1186/s12870-023-04253-4 (PMC10197446; doi:10.1186/s12870-023-04253-4)
Supplement: Supplementary file 1 — Supplementary Material 1 [file 12870_2023_4253_MOESM1_ESM.docx]

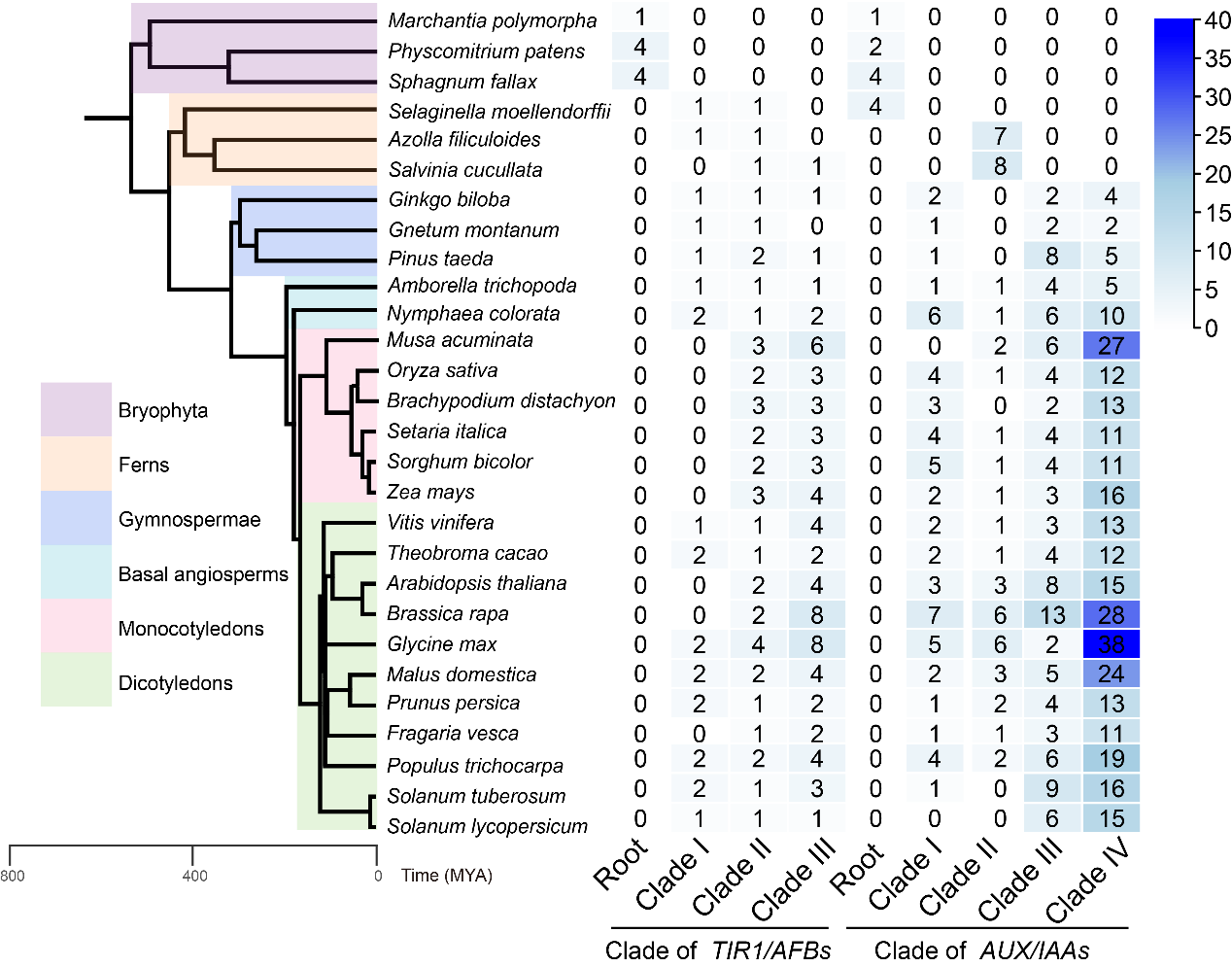


Supplementary figure 1. The distribution of *TIR1/AFBs* and *AUX/IAAs* in phylogenetic tree. Deep blue indicates a large number of values. Different background colors represent different plant groups. The clades are consistent with that in Fig. 2.


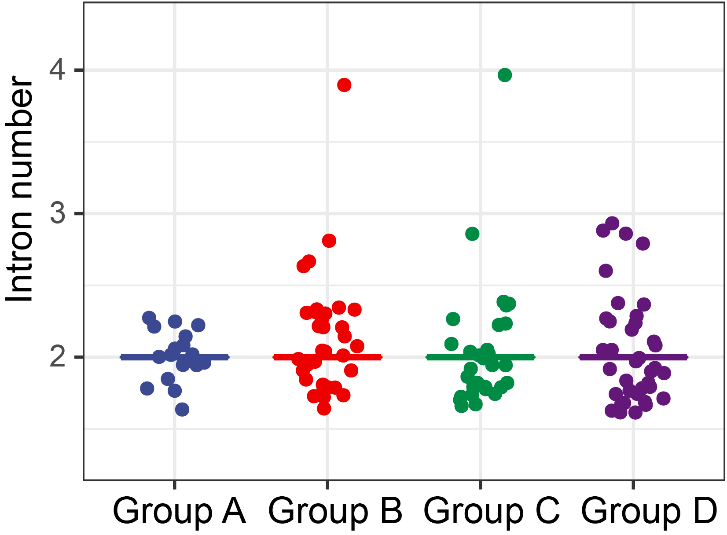


Supplementary figure 2. Intron number statistics of four groups of *TIR1/AFBs*. The groups are consistent with that in Fig. 4.


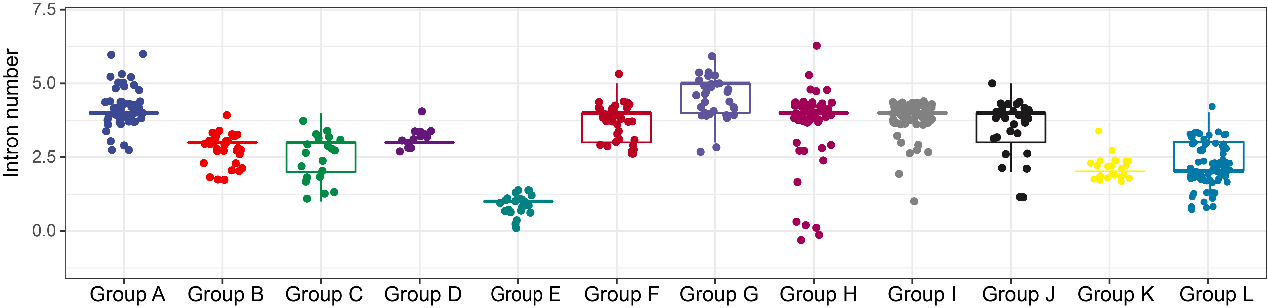


Supplementary figure 3. Intron number statistics of 12 groups of *AUX/IAAs*. The groups are consistent with that in Fig. 4.


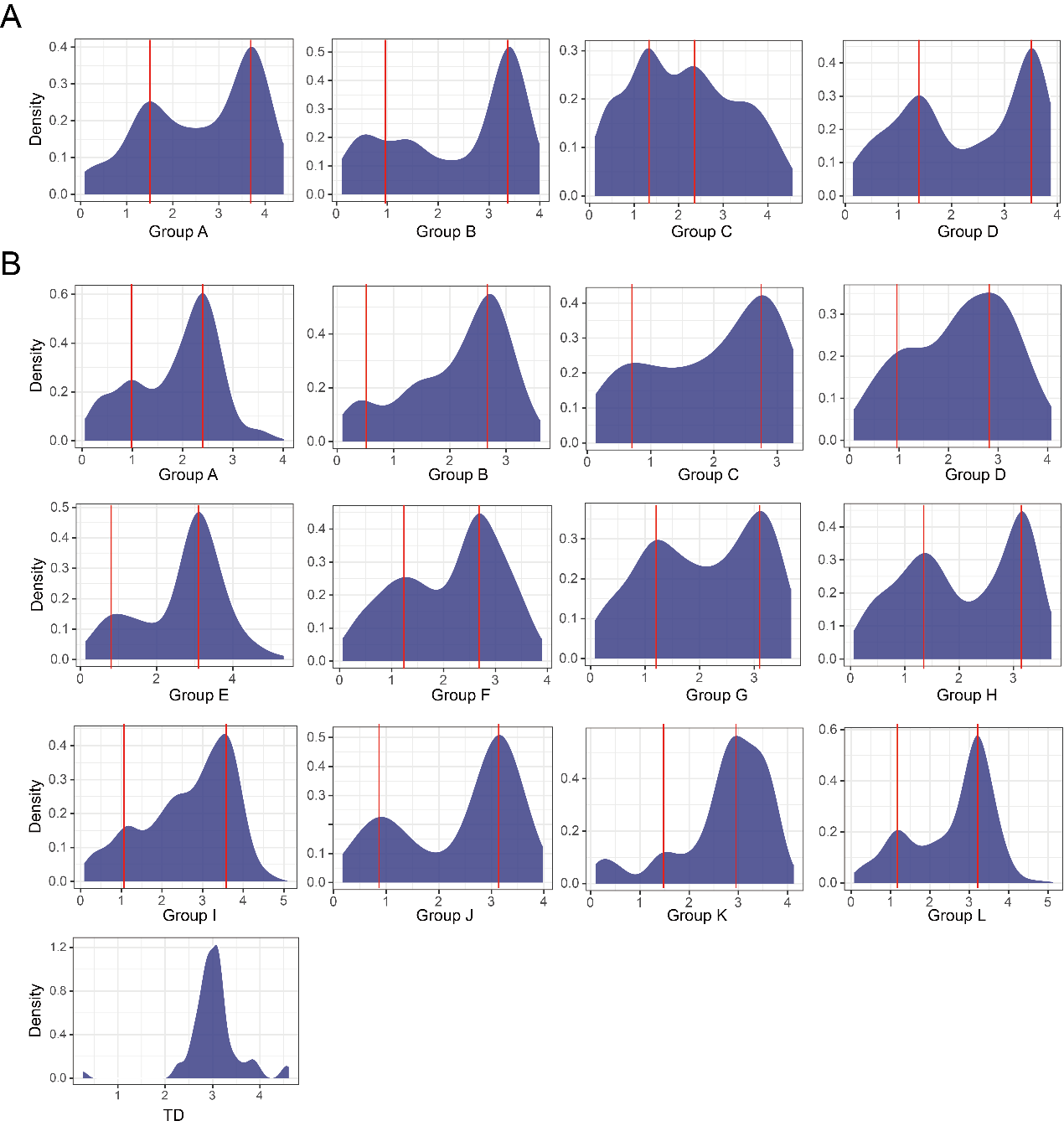


Supplementary figure 4. The gene pairs synonymous substitution (Ks) distribution of *TIR1/AFB* and *AUX/IAA* gene families. A: Gene pairs Ks distribution of *TIR1/AFB* gene families; B: Gene pairs Ks distribution of *AUX/IAA* gene families. The red line represents the whole genome duplication event. The groups are consistent with that in Fig. 4.


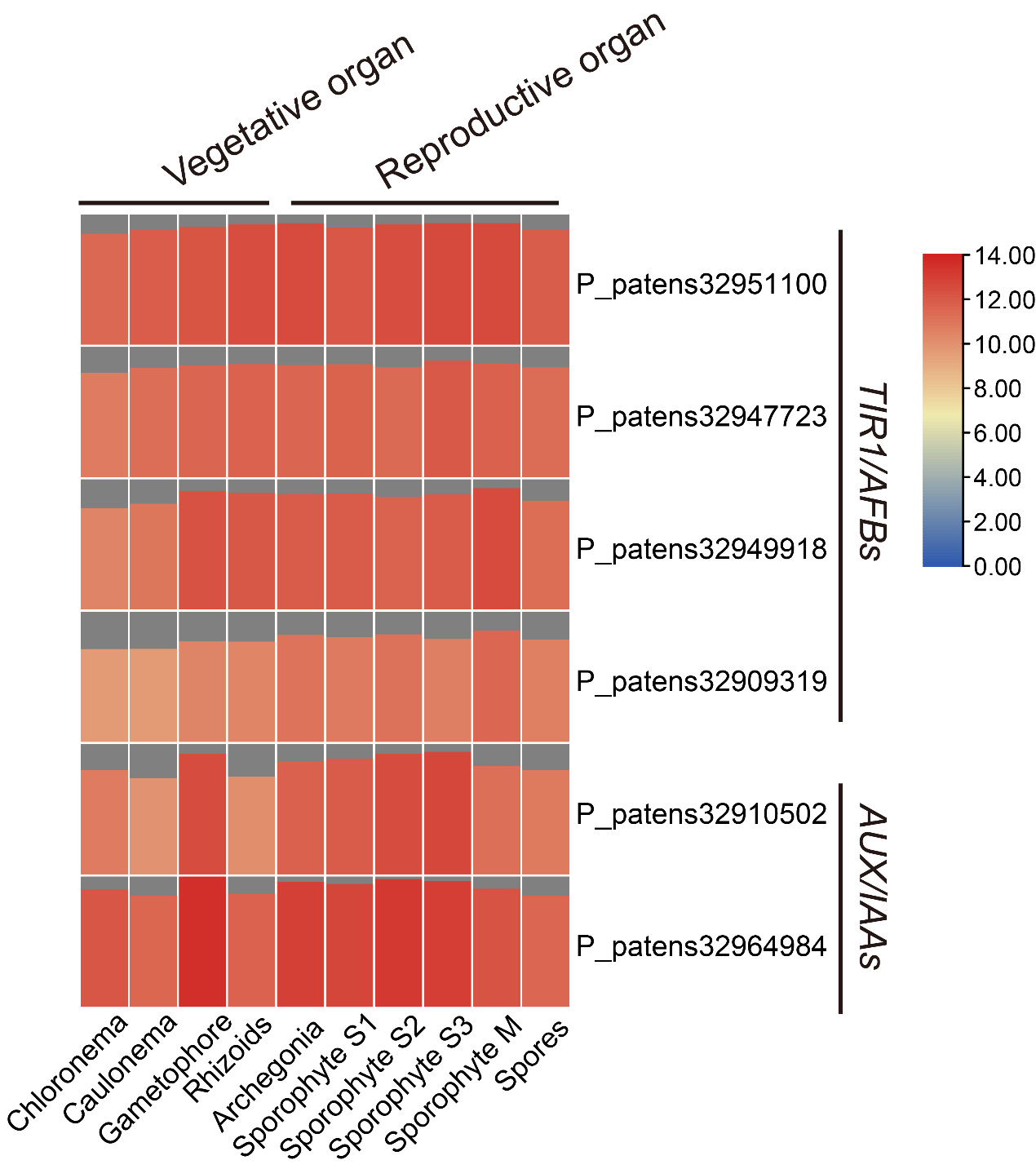


Supplementary figure 5. Expression profile of *Physcomitrium patens TIR1/AFBs* and *AUX/IAAs*.


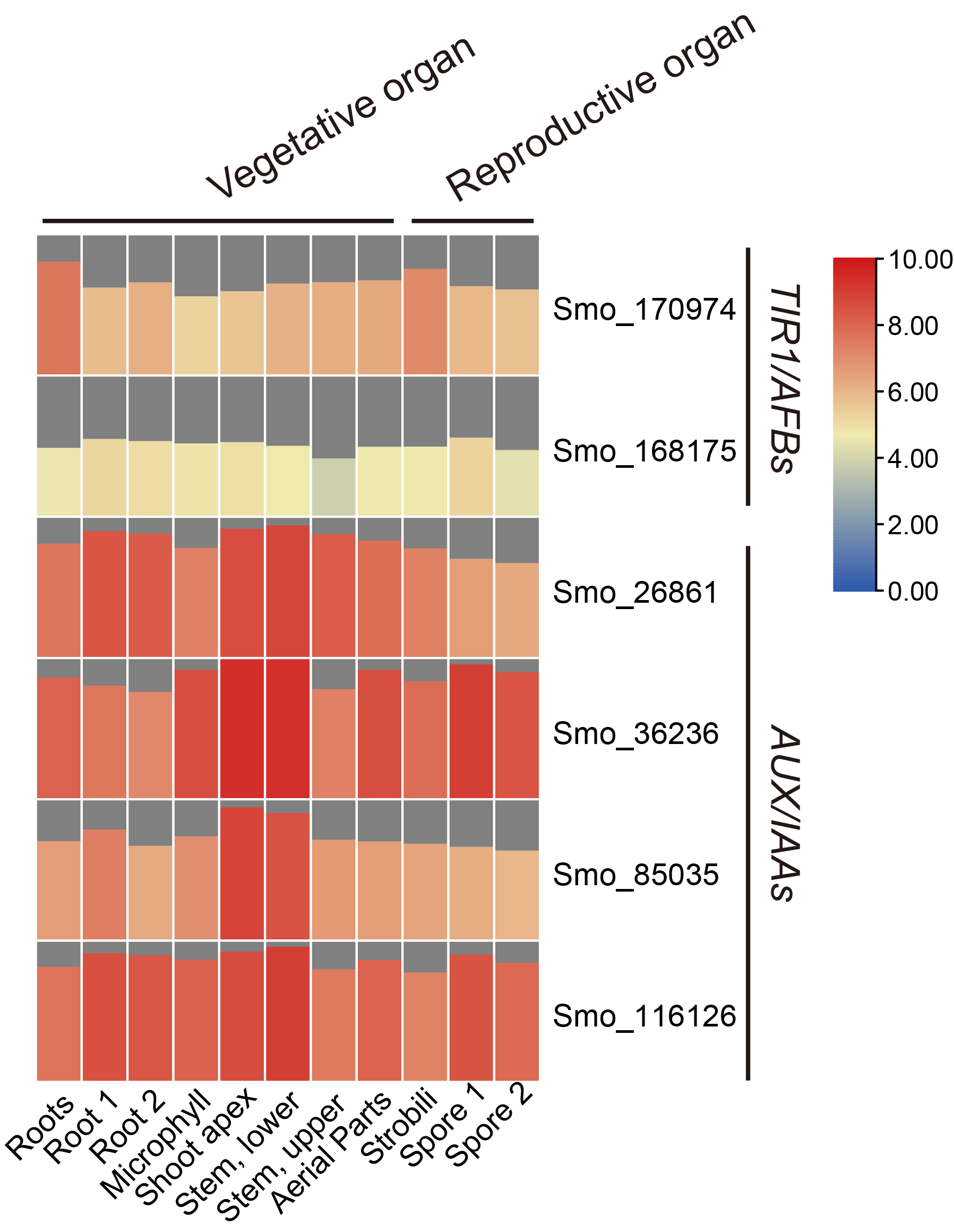


Supplementary figure 6. Expression profile of *Selaginella moellendorffii TIR1/AFBs* and *AUX/IAAs*.


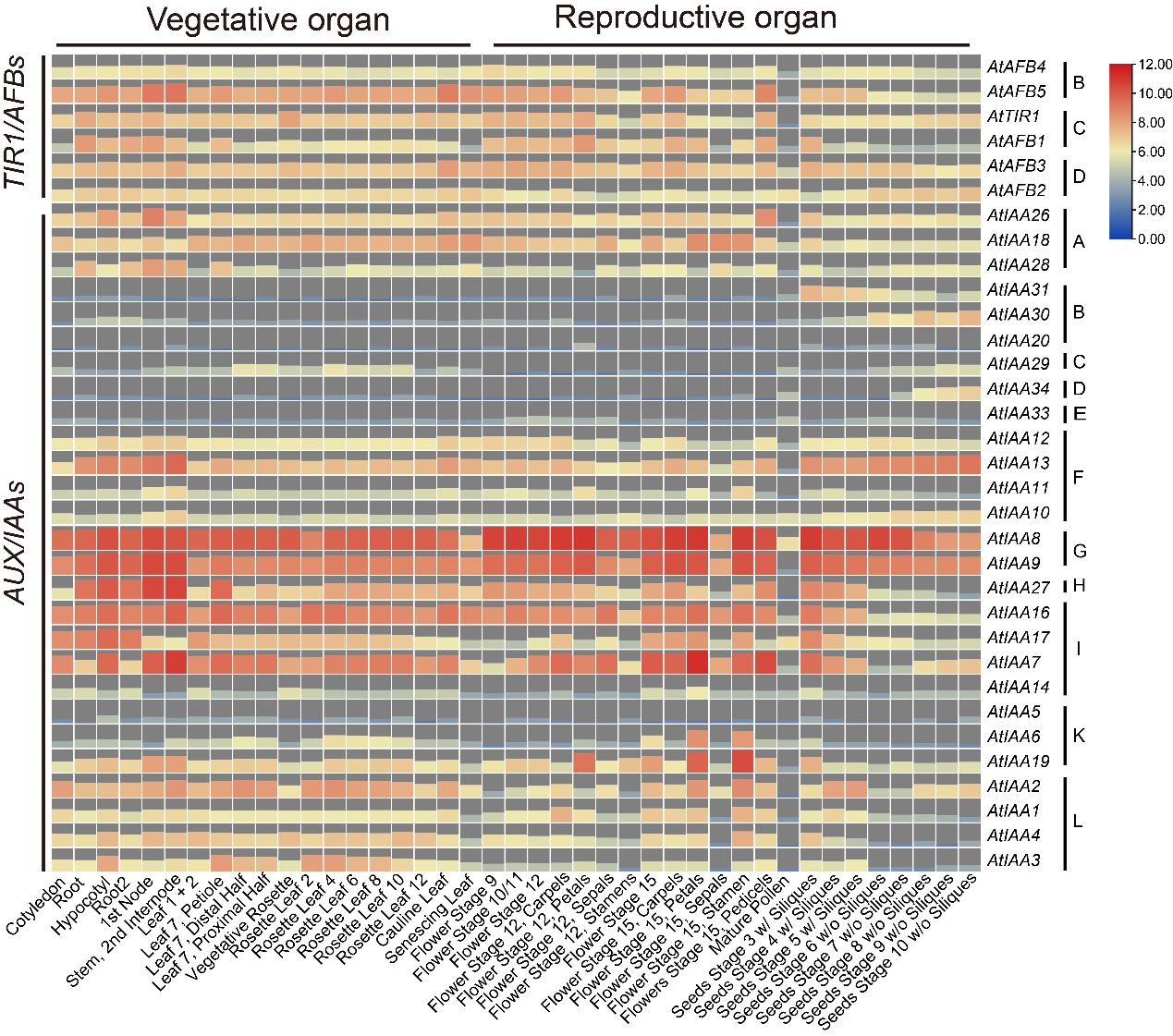


Supplementary figure 7. Expression profile of *Arabidopsis thaliana TIR1/AFBs* and *AUX/IAAs*.


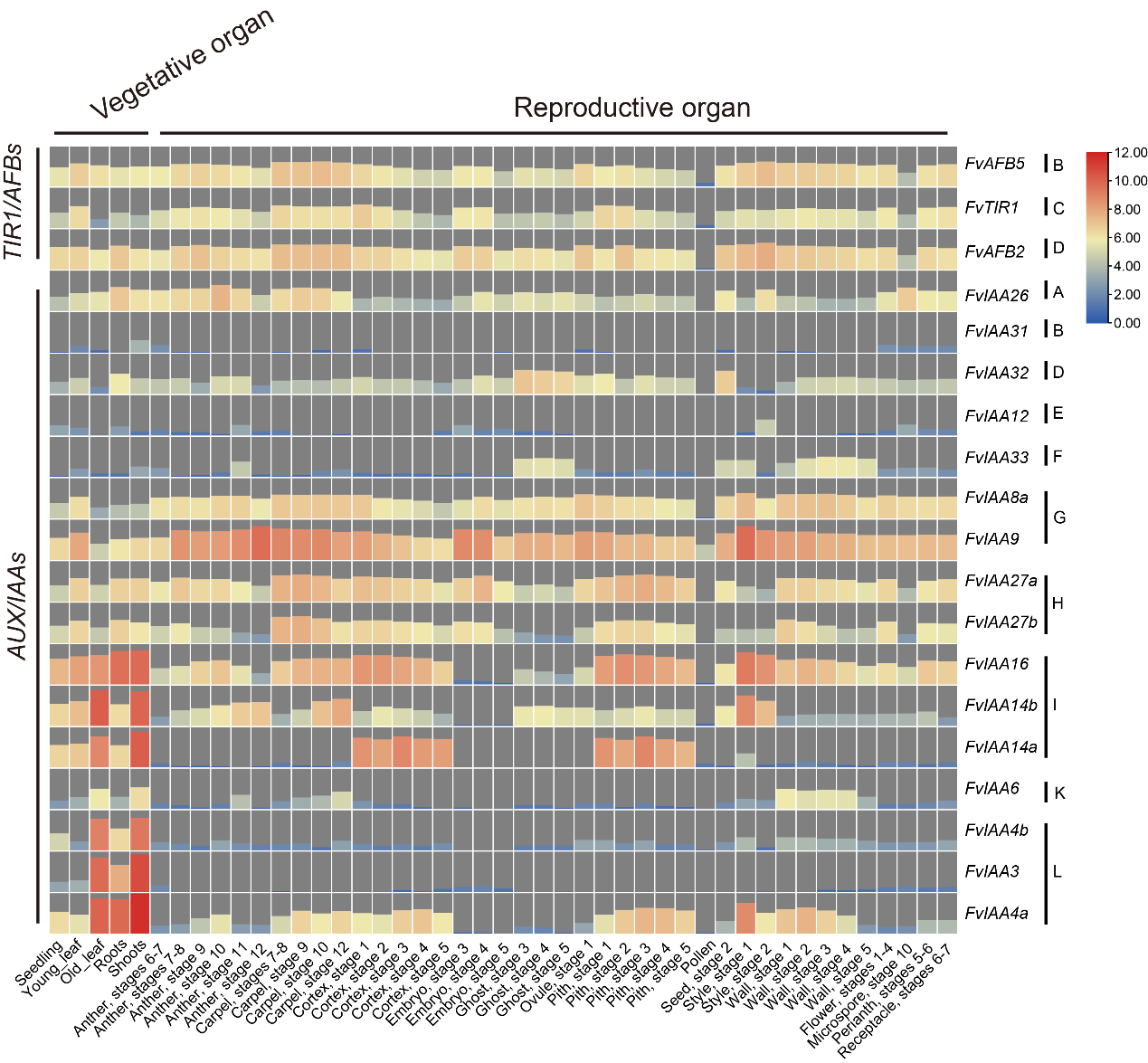


Supplementary figure 8. Expression profile of *F. vesca TIR1/AFBs* and *AUX/IAAs*.


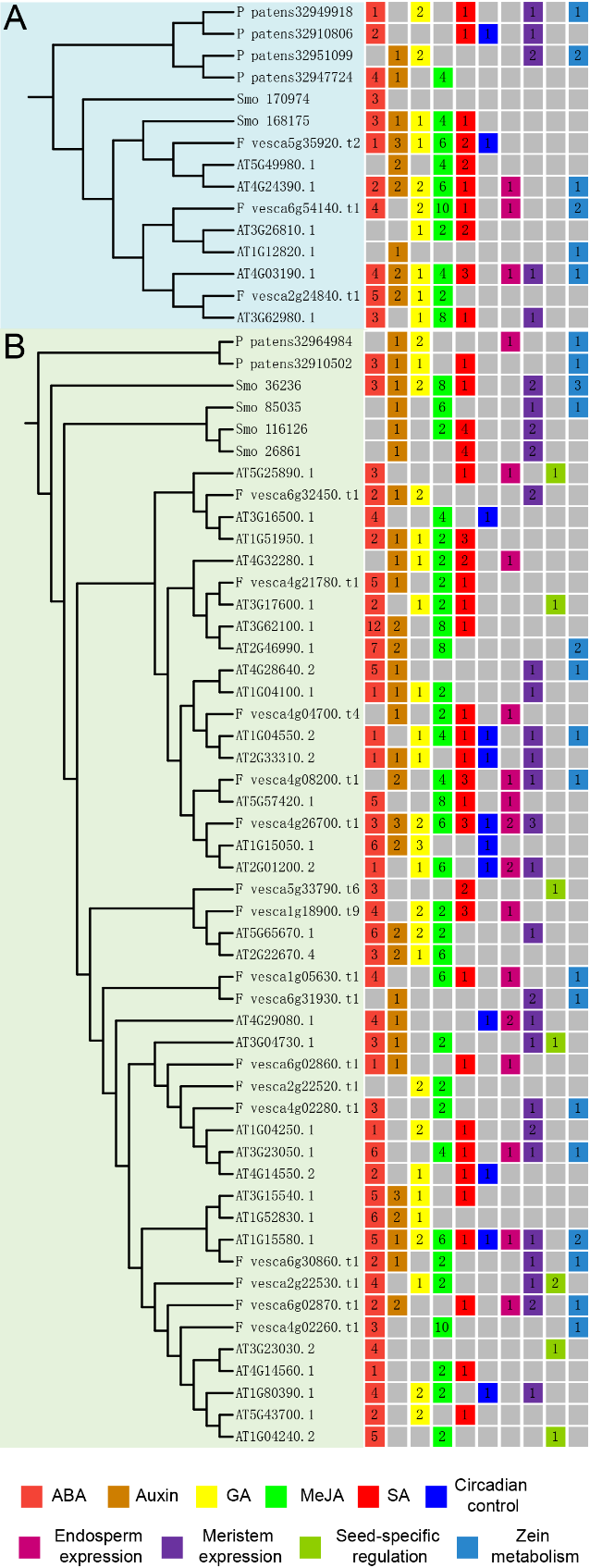


Supplementary figure 9. Detailed information of the growth and development related *cis*-elements of *TIR1/AFB* (A) *and AUX/IAA* (B) gene families in *P. Patens*, *S. moellendorfii*, *A. thaliana* *and F. vesca*. ABA, Abscisic acid; GA, Gibberellin; MeJA, Methyl jasmonate; SA, Salicylic acid. The value represents the number of *cis*-elements in the promoter.


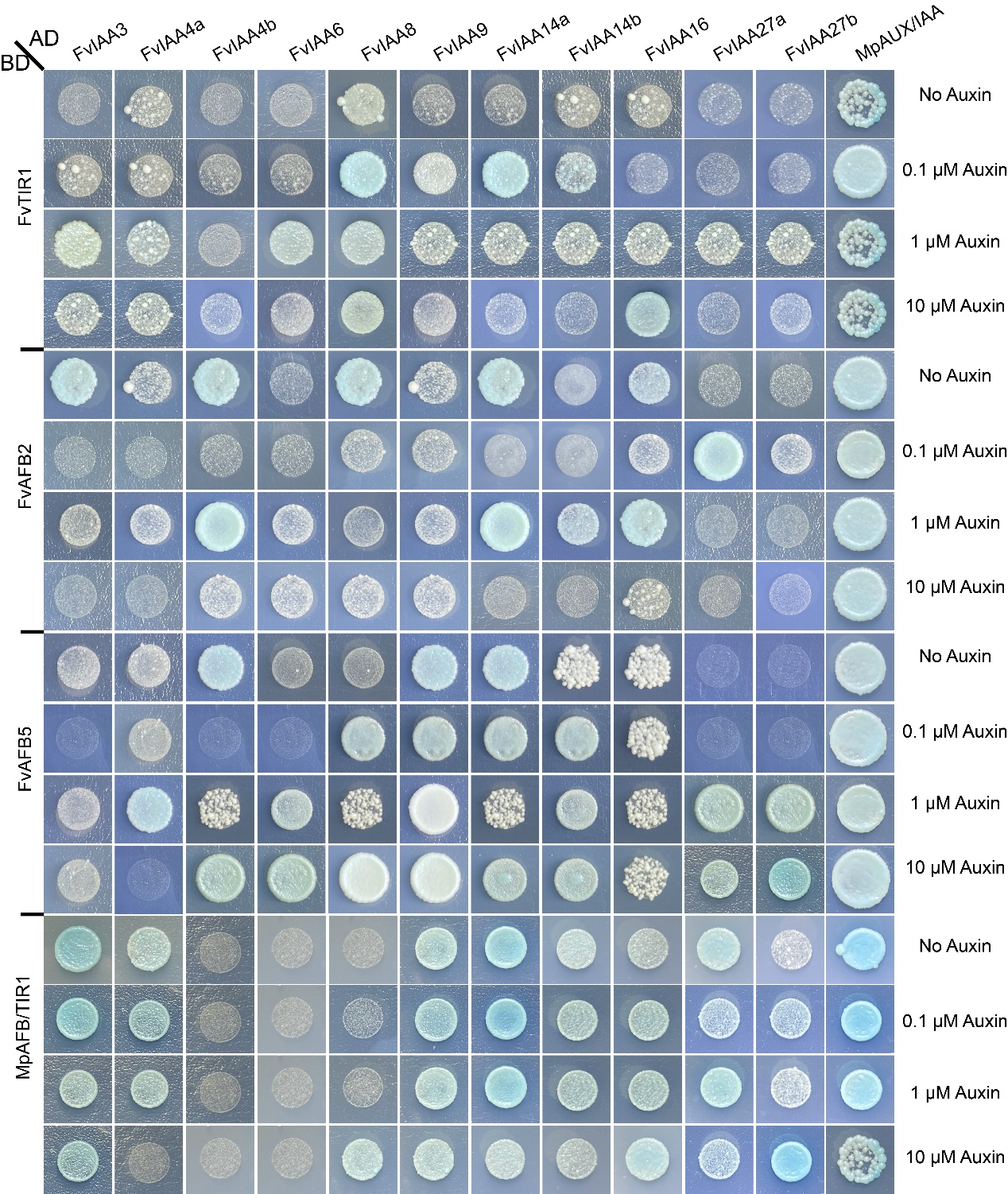


Supplementary figure 10. Interaction pattern of TIR1/AFBs and AUX/IAAs in *F. vesca* and *M. polymorpha* by the yeast two-hybrid interaction assays in SD-Leu-Trp-His+X-α-Gal medium.


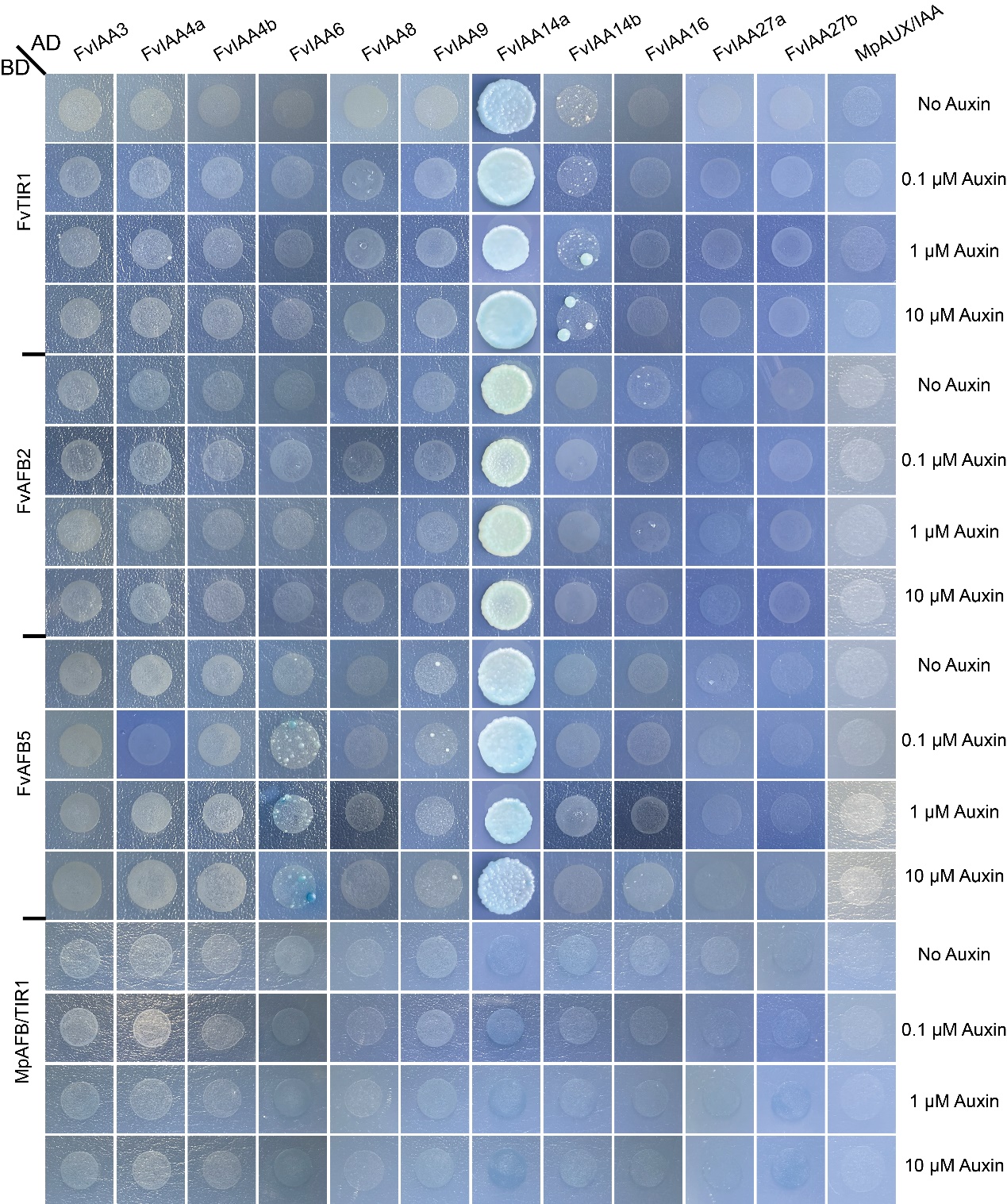


Supplementary figure 11. Interaction pattern of TIR1/AFBs and AUX/IAAs in *F. vesca* and *M. polymorpha* by the yeast two-hybrid interaction assays in SD -Leu-Trp-His-Ade +X-α-Gal medium.


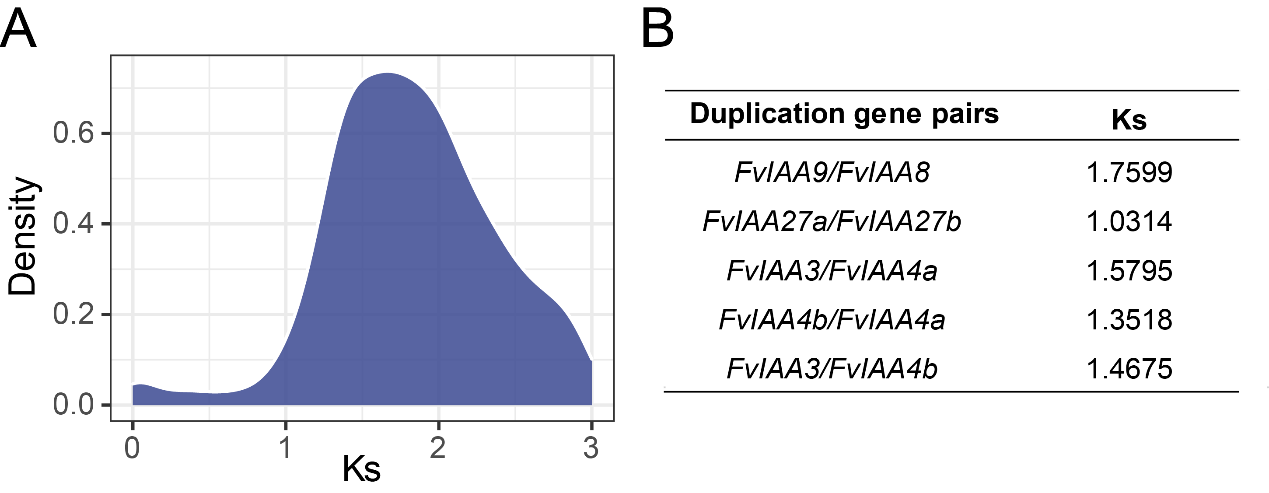


Supplementary figure 12. Ks distribution of *F. vesca* and ks value of duplication gene pairs.

Supplementary table 1. Genome data used in this study

| Species | Link and genome annotation version |
| --- | --- |
| *Porphyridium purpureum* | http://cyanophora.rutgers.edu/porphyridium |
| *Cyanidioschyzon merolae* | http://plants.ensembl.org; V1 |
| *Micromonas pusilla* | https://jgi.doe.gov; C3V2 |
| *Chlamydomonas reinhardtii* | https://jgi.doe.gov; V5 |
| *Klebsormidium nitens* | http://www.plantmorphogenesis.bio.titech.ac.jp/~algae_genome_project/klebsormidium; V1.1 |
| *Chara braunii* | https://www.ncbi.nlm.nih.gov/ |
| *Marchantia polymorpha* | http://marchantia.info; V3.1 |
| *Physcomitrium patens* | https://genome.jgi.doe.gov; V3 |
| *Sphagnum fallax* | https://phytozome.jgi.doe.gov; V0.5 |
| *Selaginella moellendorffii* | https://phytozome.jgi.doe.gov; V1.0 |
| *Azolla filiculoides* | https://www.fernbase.org; V1.1 |
| *Salvinia cucullata* | https://www.fernbase.org; V1.2 |
| *Ginkgo biloba* | https://figshare.com/articles/dataset/annotation_of_Ginkgo_biloba/14759223 |
| *Gnetum montanum* | DRYAD website |
| *Pinus taeda* | http://congenie.org/start; V1.0 |
| *Amborella trichopoda* | https://phytozome.jgi.doe.gov; V1.0 |
| *Nymphaea colorata* | https://www.ncbi.nlm.nih.gov/ |
| *Musa acuminata* | https://phytozome.jgi.doe.gov; V1 |
| *Oryza sativa* | http://rice.plantbiology.msu.edu; IRGSP-1.0 |
| *Brachypodium distachyon* | https://phytozome.jgi.doe.gov; V3.1 |
| *Setaria italica* | https://phytozome.jgi.doe.gov; V2.2 |
| *Sorghum bicolor* | https://phytozome.jgi.doe.gov; V3.1 |
| *Zea mays* | https://phytozome.jgi.doe.gov; V4 |
| *Vitis vinifera* | https://phytozome.jgi.doe.gov; V2.1 |
| *Theobroma cacao* | https://phytozome.jgi.doe.gov; V2.1 |
| *Arabidopsis thaliana* | https://www.arabidopsis.org; TAIR10 |
| *Brassica rapa* | https://phytozome.jgi.doe.gov; V1.3 |
| *Malus domestica* | https://phytozome.jgi.doe.gov; V1.1 |
| *Prunus persica* | Genome Database for Rosaceae, V2.0.a1 |
| *Fragaria vesca* | Genome Database for Rosaceae, V4.2 |
| *Glycine max* | https://phytozome.jgi.doe.gov; V4.1 |
| *Populus trichocarpa* | https://phytozome.jgi.doe.gov; V4.1 |
| *Solanum tuberosum* | https://phytozome.jgi.doe.gov; V6.1 |
| *Solanum lycopersicum* | https://phytozome.jgi.doe.gov; V4.0 |

Supplementary table 2. The primer used in this study.

| Name of primers | Sequences (5’-3’) | Annotation |
| --- | --- | --- |
| FvTIR1-F | TGGCCATGGAGGCCGAATTCATGCTGAGAATGGCGAGCTCGTTC | Vectors construction |
| FvTIR1-R | CGCTGCAGGTCGACGGATCCTCAAGTGAGCCTCACTGCAGAGTG |  |
| FvAFB2-F | TGGCCATGGAGGCCGAATTCATGAATTATTTCCCAGACGAGG |  |
| FvAFB2-R | CGCTGCAGGTCGACGGATCCCTACAGAGTCCACACATACT |  |
| FvAFB5-F | TGGCCATGGAGGCCGAATTCATGGGAGACGACCCTTCTTCGT |  |
| FvAFB5-R | CGCTGCAGGTCGACGGATCCCTACAGGATATCAACAAACTTTGG |  |
| MpTIR1-F | TGGCCATGGAGGCCGAATTCATGCCCTCTCCCTTTCCTGACGA |  |
| MpTIR1-R | CGCTGCAGGTCGACGGATCCTCATTGTGCTATTTCGACA |  |
| FvIAA14a-F | CCATGGAGGCCAGTGAATTCATGGAAGTTGTTATCCGGAAGA |  |
| FvIAA14a-R | AGCTCGAGCTCGATGGATCCTCAGCTTCTGTTCTTGCATTTC |  |
| FvIAA14b-F | CCATGGAGGCCAGTGAATTCATGGAGACAGAGCTCAGGCTAG |  |
| FvIAA14b-R | AGCTCGAGCTCGATGGATCCTCAGCTTCTGTTCTTGCATTTC |  |
| FvIAA16-F | CCATGGAGGCCAGTGAATTCATGACTAGTACTGTAGCCATGG |  |
| FvIAA16-R | AGCTCGAGCTCGATGGATCCTCAACTTCTGTTCTTGAACTTC |  |
| FvIAA27a-F | CCATGGAGGCCAGTGAATTCATGTCTATGTCCTTGGAGCATG |  |
| FvIAA27a-R | AGCTCGAGCTCGATGGATCCCTAATTACTATTTTTGCACTTCTG |  |
| FvIAA27b-F | CCATGGAGGCCAGTGAATTCATGTCTGTACCACTGGAACATG |  |
| FvIAA27b-R | AGCTCGAGCTCGATGGATCCCTAGTTGCGGTTCCTGCACTTC |  |
| FvIAA3-F | CCATGGAGGCCAGTGAATTCATGGAAAGCAAGGGCTATGAG |  |
| FvIAA3-R | AGCTCGAGCTCGATGGATCCTCATACACCACAACCCAAGCCT |  |
| FvIAA4a-F | CCATGGAGGCCAGTGAATTCATGGCATTCCAAGCAGAAGATC |  |
| FvIAA4a-R | AGCTCGAGCTCGATGGATCCTCATACCGCACAACCTAAGCC |  |
| FvIAA4b-F | CCATGGAGGCCAGTGAATTCATGGAAGGGTCGGTGAGCTA |  |
| FvIAA4b-R | AGCTCGAGCTCGATGGATCCTCAGAGACAAGCCAGTCCTCTA |  |
| FvIAA6-F | CCATGGAGGCCAGTGAATTCATGGAGAAAGAAGGTCTAGG |  |
| FvIAA6-R | AGCTCGAGCTCGATGGATCCTTATTTAGGCTCGTCTTTCATC |  |
| FvIAA8-F | CCATGGAGGCCAGTGAATTCATGTCACAACCACAGGTCGGT |  |
| FvIAA8-R | AGCTCGAGCTCGATGGATCCCTAGTTCCTGTTCCTGCACTT |  |
| FvIAA9-F | CCATGGAGGCCAGTGAATTCATGTCTCCACCACTGCTGGGTG |  |
| FvIAA9-R | AGCTCGAGCTCGATGGATCCCTAGTTCCGGATCTTGGATTTC |  |
| MpIAA-F | CCATGGAGGCCAGTGAATTCATGAGTCAAAATGCGCATGCTG |  |
| MpIAA-R | AGCTCGAGCTCGATGGATCCTCACACGTTCGGTTGAGTCGTCTTG |  |

Supplementary table 3. All amplified genes in this experiment.

| Original ID | ID for this study | Annotation | Genome annotation version |
| --- | --- | --- | --- |
| FvH4_1g18900.t9 | F_vesca1g18900.t9 | *FvIAA8* | Genome Database for Rosaceae, V4.2 |
| FvH4_5g33790.t6 | F_vesca5g33790.t6 | *FvIAA9* |  |
| FvH4_1g05630.t1 | F_vesca1g05630.t1 | *FvIAA27a* |  |
| FvH4_6g31930.t1 | F_vesca6g31930.t1 | *FvIAA27b* |  |
| FvH4_2g22520.t1 | F_vesca2g22520.t1 | *FvIAA14b* |  |
| FvH4_4g02280.t1 | F_vesca4g02280.t1 | *FvIAA14a* |  |
| FvH4_6g02860.t1 | F_vesca6g02860.t1 | *FvIAA16* |  |
| FvH4_6g30860.t1 | F_vesca6g30860.t1 | *FvIAA6* |  |
| FvH4_2g22530.t1 | F_vesca2g22530.t1 | *FvIAA4b* |  |
| FvH4_4g02260.t1 | F_vesca4g02260.t1 | *FvIAA3* |  |
| FvH4_6g02870.t1 | F_vesca6g02870.t1 | *FvIAA4a* |  |
| FvH4_5g35920.t2 | F_vesca5g35920.t2 | *FvAFB5* |  |
| FvH4_2g24840.t1 | F_vesca2g24840.t1 | *FvTIR1* |  |
| FvH4_6g54140.t1 | F_vesca6g54140.t1 | *FvAFB2* |  |
| Mapoly0035s0062.1p | Mapoly0035s0062.1p | *MpAUX/IAA* | http://marchantia.info; V3.1 |
| Mapoly0034s0017.1p | Mapoly0034s0017.1p | *MpTIR1/AFB* |  |
